# Coordination Polymers Assembled from Flexible Tricarboxylate Linkers: Hydrothermal Synthesis, Structural Diversity, and Catalytic Features

**DOI:** 10.1021/acs.inorgchem.5c05274

**Published:** 2026-02-07

**Authors:** Wei Dou, Beining Shi, Xiaoxiang Fan, Jinzhong Gu, Marina V. Kirillova, Alexander M. Kirillov

**Affiliations:** † State Key Laboratory of Natural Product Chemistry, 12426 College of Chemistry and Chemical Engineering, Lanzhou University, Lanzhou 730000, People’s Republic of China; ‡ Nuclear Power Institute of China, Chengdu 610041, People’s Republic of China; § MINDlab: Molecular Design & Innovation Laboratory, Centro de Química Estrutural, Institute of Molecular Sciences, Departamento de Engenharia Química, Instituto Superior Técnico, 72971 Universidade de Lisboa, Av. Rovisco Pais, Lisbon 1049-001, Portugal

## Abstract

The molecular design of coordination polymers (CPs) and metal–organic frameworks (MOFs) has attracted increasing attention in the areas of inorganic chemistry and functional materials. In this study, a new series of 2D CPs and 3D MOFs was hydrothermally assembled from metal­(II) chlorides and 2,2’-((4-carboxy-1,2-phenylene)*bis*(oxy))­diacetic acid (H_3_cpbda) as a flexible and little-explored tricarboxylate linker. Additionally, several types of aromatic *N*,*N*-donor auxiliary ligands were used to promote crystallization, namely, 1,10-phenanthroline (phen), 4,4′-bipyridine (bipy), bis­(4-pyridyl)­amine (bpa), 1,2-di­(4-pyridly)­ethylene (dpey), or 1,2-di­(4-pyridly)­ethane (dpea). The obtained products were fully characterized and identified as [M_3_(μ_6_-cpbda)_2_(phen)_2_]_*n*_·4*n*H_2_O (M = Zn (**1**), Cd (**2**)), [Co_3_(μ_5_-cpbda)_2_(μ-bipy)_2_]_*n*_·2*n*H_2_O (**3**), [Zn_3_(μ_5_-cpbda)_2_(μ-bipy)_2_]_*n*_ (**4**), [Zn­(μ_3_-cpbda)­(Hbpa)]_*n*_·4*n*H_2_O (**5**), [Zn_4_(μ_3_-cpbda)_2_(μ-OH)_2_(μ-dpey)_3_(H_2_O)_2_]_*n*_·2*n*H_2_O (**6**), [Co_3_(μ_4_-cpbda)_2_(μ-dpey)_3_]_*n*_·2*n*H_2_O (**7**), and [Ni_3_(μ_4_-cpbda)_2_(μ-dpea)_3_]_*n*_·2*n*H_2_O (**8**). Their structural and topological features were also explored, allowing us to identify a diversity of 2D and 3D coordination networks. Remarkably, Zn-based coordination polymers **5** and **6** revealed a high catalytic activity and reusability in the condensation reaction between benzaldehyde and malononitrile (or ethyl cyanoacetate), leading to almost quantitative product yields (99%) under optimized conditions. The present work contributes to widening the family of CPs/MOFs assembled from flexible polycarboxylate linkers and highlights a promising application of these compounds in heterogeneous catalysis.

## Graphical abstract

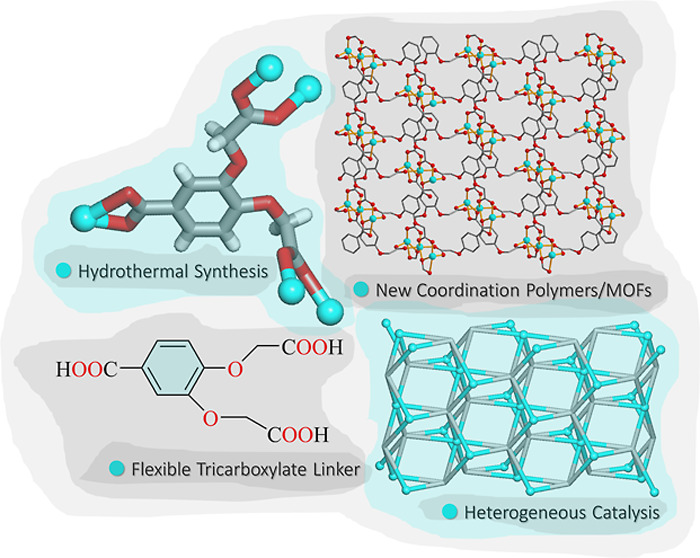

## Introduction

Coordination polymers (CPs) and metal–organic frameworks (MOFs) have attracted extensive attention owing to their structural diversity and tunability, which arise from the versatile combination of organic linkers and metal nodes.^1–5^ The rational design of functional CPs has become a central theme in inorganic chemistry and materials science, driven by the broad potential applications of these materials in gas storage and separation,^6–11^ sensing,^12–14^ magnetism,^15–18^ biomedical areas,^19–21^ and catalysis.^22–29^

Benefiting from facile synthesis, tunable structures, and notable chemical stability, CPs and MOFs represent an attractive platform for the development of advanced functional materials. Among these, CP-based heterogeneous catalysts have received particular interest due to their notable activity, selectivity, and recyclability.^22–32^ In particular, the condensation of aldehydes with dinitriles can be employed as a model reaction to assess the catalytic performance of new CPs, owing to a simplicity of this transformation, its importance in forming C–C bonds, and its ability to proceed via both Lewis acid and base pathways.^33–36^

As a continuation of our previous studies on functional CPs derived from commercially available carboxylic acid linkers,^37–39^ we have now investigated the flexible tricarboxylate ligand H_3_cpbda (2,2’-((4-carboxy-1,2-phenylene)*bis*(oxy))­diacetic acid, Scheme 1) as an underexplored linker to construct coordination polymers.^40^ H_3_cpbda was chosen owing to its six potential coordination sites and the presence of one _Ph_COOH and two more flexible _Ph_OCH_2_COOH functionalities, which enable diverse coordination modes. In this study, we report the hydrothermal synthesis and characterization of eight new CPs and MOFs assembled from metal­(II) salts, H_3_cpbda, and various *N*,*N*-donor auxiliary ligands acting as mediators of crystallization (Scheme 1). These products exhibit diverse structures, including two-dimensional layers (**1**, **2**, **5**, and **6**) and three-dimensional frameworks (**3**, **4**, **7**, and **8**), formulated as [M_3_(μ_6_-cpbda)_2_(phen)_2_]_*n*_·4*n*H_2_O (M = Zn­(**1**), Cd­(**2**)), [Co_3_(μ_5_-cpbda)_2_(μ-bipy)_2_]_*n*_·2*n*H_2_O (**3**), [Zn_3_(μ_5_-cpbda)_2_(μ-bipy)_2_]_*n*_ (**4**), [Zn­(μ_3_-cpbda)­(Hbpa)]_*n*_·4*n*H_2_O (**5**), [Zn_4_(μ_3_-cpbda)_2_(μ-OH)_2_(μ-dpey)_3_(H_2_O)_2_]_*n*_·2*n*H_2_O (**6**), [Co_3_(μ_4_-cpbda)_2_(μ-dpey)_3_]_*n*_·2*n*H_2_O (**7**), and [Ni_3_(μ_4_-cpbda)_2_(μ-dpea)_3_]_*n*_·2*n*H_2_O (**8**). Structural and topological features as well as catalytic performance of the obtained products in the condensation reaction between benzaldehyde (model substrate) and nitrile derivatives (malononitrile or ethyl cyanoacetate) are described below. This work expands the family of CPs/MOFs constructed from flexible polycarboxylate linkers and underscores the promising potential of these materials in heterogeneous catalysis.

**Scheme 1:**
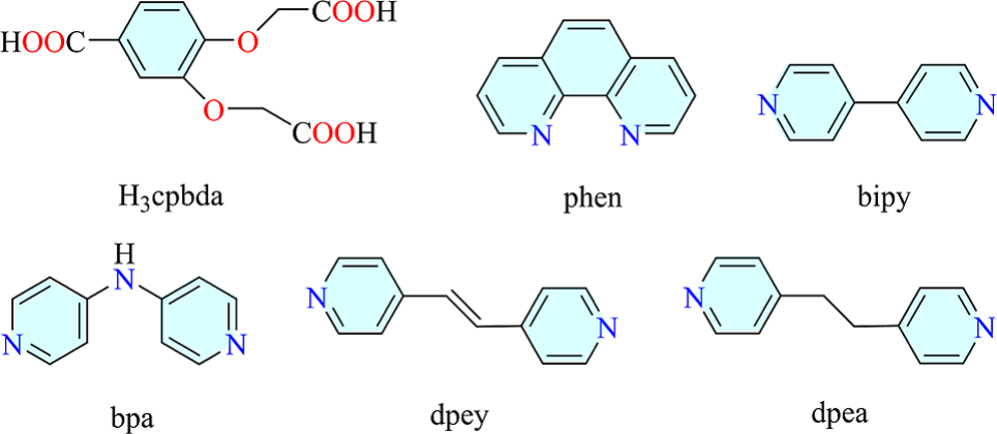
Structures of H_3_cpbda and Auxiliary Ligands

## Experimental Section

### Synthesis

All chemicals were obtained from commercial suppliers. Instruments and full synthetic procedures are described in the Supporting Information. The hydrothermal syntheses of **1**–**8** were based on the reactions of metal­(II) chlorides in water with H_3_cpbda as the primary building block, different *N*,*N*-donor auxiliary ligands (phen, bipy, bpa, dpey, and dpea), and sodium hydroxide as a deprotonating agent. The reactions were performed at 160 °C for 3 days in a Teflon-lined stainless-steel autoclave (Table 1). The detailed synthetic procedures and analytical data for compounds **1**–**8** are given in Supporting Information.

**Table 1:** Molecular Formulas and Reaction Conditions for Compounds **1**–**8** ^a^

molecular formula	metal(II) salt	auxiliary ligand (AL)	dimensionality	topology
[Zn_3_(μ_6_-cpbda)_2_(phen)_2_]_*n*_·4*n*H_2_O (**1**)	ZnCl_2_	phen	2D	3,6,6L3
[Cd_3_(μ_6_-cpbda)_2_(phen)_2_]_*n*_·4*n*H_2_O (**2**)	CdCl_2_·H_2_O	phen	2D	3,6,6L3
[Co_3_(μ_5_-cpbda)_2_(μ-bipy)_2_]_*n*_·2*n*H_2_O (**3**)	CoCl_2_·6H_2_O	bipy	3D	4,5,6T22
[Zn_3_(μ_5_-cpbda)_2_(μ-bipy)_2_]_*n*_ (**4**)	ZnCl_2_	bipy	3D	4,5,6T22
[Zn(μ_3_-cpbda)(Hbpa)]_*n*_·4*n*H_2_O (**5**)	ZnCl_2_	bpa	2D	fes
[Zn_4_(μ_3_-cpbda)_2_(μ-OH)_2_(μ-dpey)_3_(H_2_O)_2_]_*n*_·2*n*H_2_O (**6**)	ZnCl_2_	dpey	2D	3,6L77
[Co_3_(μ_4_-cpbda)_2_(μ-dpey)_3_]_*n*_·2*n*H_2_O (**7**)	CoCl_2_·6H_2_O	dpey	3D	new
[Ni_3_(μ_4_-cpbda)_2_(μ-dpea)_3_]_*n*_·2*n*H_2_O (**8**)	NiCl_2_·6H_2_O	dpea	3D	new

a Hydrothermal synthesis in a Teflon-lined stainless-steel reactor (25 mL volume), H_2_O (10 mL), 3 days, 160 °C, MCl_2_:H_3_cpbda:AL:NaOH molar ratio 3:2:3:6.

### X-ray Crystallography

A diffractometer with graphite-monochromated MoK_α_ radiation (Bruker APEX-II CCD; λ = 0.71073 Å) was used to obtain the crystallographic data for **1**–**8**. The structures were determined through direct approaches and refined using full-matrix least-squares on *F* ^2^ with the SHELXS-97 and SHELXL-97 programs.^41^ Carbon, oxygen, and nitrogen atoms were refined anisotropically using full-matrix least-squares on *F* ^2^, while hydrogen atoms were added to calculated positions. Summary of crystallographic data is given in Table 2. The main bonding parameters are listed in Tables S1 and S2. CCDC 2500238–2500245 contain the crystallographic data for **1**–**8**. Topological analyses of metal–organic networks were performed on ToposPro software, following the concept of an underlying net. Such nets were generated by reducing all the bridging ligands to centroids and preserving the connectivity of metal centers and linkers.^42,43^

**Table 2:** Summary of Structural Data for Compounds **1**–**8**

compound	**1**	**2**	**3**	**4**
chemical formula	C_46_H_38_Zn_3_N_4_O_20_	C_46_H_38_Cd_3_N_4_O_20_	C_42_H_34_Co_3_N_4_O_18_	C_42_H_30_Zn_3_N_4_O_16_
formula weight	1162.92	1304.00	1059.52	1042.87
crystal system	monoclinic	orthorhombic	monoclinic	monoclinic
space group	*C*2/*c*	*Fdd*2	*P*2_1_/*n*	*P*2_1_/*n*
*a*/Å	16.6192(2)	52.2070(5)	9.1440(2)	9.13680(10)
*b*/Å	11.36420(10)	16.88528(17)	17.9979(3)	18.1144(2)
*c*/Å	25.7744(2)	11.55829(13)	12.9427(3)	13.0076(2)
α/°	90	90	90	90
β/°	97.6070(10)	90	109.888(3)	109.1030(10)
γ/°	90	90	90	90
*V*/Å^3^	4825.01(8)	10188.97(18)	2002.98(8)	2034.30(5)
*T*/K	301(2)	150(2)	150(2)	303(2)
*Z*	4	8	2	2
*D*_*c*_/g cm^–3^	1.601	1.700	1.757	1.702
μ/mm^–1^	2.471	10.634	10.401	2.775
*F*(000)	2368	5168	1078	1056
refl. measured	5002	4313	3702	3746
unique refl. (*R*_int_)	4767 (0.0325)	4276 (0.0533)	3408 (0.0461)	3505 (0.0269)
GOF on *F* ^2^	1.074	1.010	1.049	1.048
*R*_1_[*I* > 2σ(*I*)]^*a*^	0.0384	0.0368	0.0541	0.0320
*wR*_2_[*I* > 2σ(*I*)]^*b*^	0.1042	0.0975	0.1439	0.0787

compound	**5**	**6**	**7**	**8**
chemical formula	C_21_H_25_ZnN_3_O_12_	C_58_H_54_Zn_4_N_6_O_22_	C_58_H_48_Co_3_N_6_O_18_	C_58_H_54_Ni_3_N_6_O_18_
formula weight	576.77	1448.55	1293.81	1299.20
crystal system	orthorhombic	triclinic	monoclinic	monoclinic
space group	*Pbca*	*P*1̅	*P*2_1_/*n*	*P*2_1_/*n*
*a*/Å	15.6879(3)	12.15910(10)	9.6461(2)	9.7380(2)
*b*/Å	14.2493(4)	14.2152(2)	20.2744(4)	20.3685(3)
*c*/Å	21.5236(5)	17.3810(2)	13.7233(2)	13.7214(2)
α/°	90	73.4040(10)	90	90
β/°	90	88.0500(10)	105.222(2)	105.191(2)
γ/°	90	79.9180(10)	90	90
*V*/Å^3^	4811.4(2)	2834.19(6)	2589.69(9)	2626.52(8)
*T*/K	303(2)	150(2)	150(2)	294.9(4)
*Z*	8	2	2	2
*D*_*c*_/g cm^–3^	1.394	1.697	1.659	1.643
μ/mm^–1^	1.852	2.691	8.183	1.983
*F*(000)	2064	1480	1326	1344
refl. measured	4418	10680	4887	5227
unique refl. (*R*_*int*_)	3410 (0.0491)	9798 (0.0255)	4459 (0.0352)	4540 (0.0364)
GOF on *F* ^2^	1.019	1.020	1.006	1.172
*R*_1_[*I* > 2σ(*I*)]^*a*^	0.0581	0.0320	0.0421	0.0661
*wR*_2_[*I* > 2σ(*I*)]^*b*^	0.1666	0.0908	0.1051	0.0761

### Catalytic Studies

In a typical protocol, benzaldehyde (0.50 mmol), malononitrile (1.0 mmol) or ethyl cyanoacetate (1.0 mmol), and catalyst (typically 2.0 mol %) were combined in CH_3_OH (1.0 mL), and the obtained suspension was stirred for 10–60 min at 25 °C. The catalyst was then isolated by centrifugation and the filtrate was evaporated under reduced pressure, resulting in crude solid. This was dissolved in CDCl_3_ and analyzed by ^1^H NMR spectroscopy (JNM ECS 400 M spectrometer) for product quantification (further details are given in SI, Figures S3 and S4). In catalyst recycling experiments, the catalyst was removed by centrifugation, washed with CH_3_OH, dried at 25 °C, and reused in subsequent catalytic tests. Effects of catalyst and solvent as well as substrate scope with other aldehydes (Tables S3 and S6) were investigated following the above-described procedure.

## Results and Discussion

### Hydrothermal Synthesis of Compounds **1**–**8**

To explore 2,2’-((4-carboxy-1,2-phenylene)*bis*(oxy))­diacetic acid (H_3_cpbda) as a flexible linker for the design of new coordination polymers, we performed a number of synthetic attempts under hydrothermal conditions. The reaction mixtures in H_2_O were composed of metal­(II) chloride (Zn, Co, Ni, or Cd chloride), H_3_cpbda as the main linker, sodium hydroxide as a deprotonating agent, and an auxiliary *N*,*N*-donor ligand (phen, bipy, bpa, dpey, or dpea) that also acted as a mediator of crystallization. The mixtures were heated at 160 °C for 3 days, followed by a slow cooling to room temperature and a crystallization of products.

Different metal­(II) precursors were screened to explore structural diversity and catalytic properties of metal ions, considering that Zn and Cd may provide coordinatively unsaturated metal centers and favor flexible nodes for framework assembly, while Co and Ni provide open d-shell centers with a typical octahedral environment. Sodium hydroxide was chosen as a very common deprotonating agent for carboxylate-based linkers in aqueous media, which proved critical for the hydrothermal formation of phase-pure CPs/MOFs,^37–39^ also considering the presence in the reaction system of *N*,*N*-donor auxiliary ligands that can act as milder organic bases.

The successful reactions led to the formation of stable and crystalline solids that were isolated in yields varying from 33 to 47%. The obtained CPs/MOFs were characterized by standard techniques, including single-crystal X-ray diffraction, and their molecular formulas were established (Table 1). The purity of compounds **1**–**8** was confirmed by powder X-ray diffraction (PXRD) analyses. The experimentally obtained diffractograms for bulk samples closely match the patterns simulated from the single-crystal X-ray data (Figure S2). The structural variability among **1**–**8** is associated with the nature of metal­(II) centers and coordination modes of the main linker (Scheme 2), and the presence of different auxiliary ligands.

**Scheme 2:**
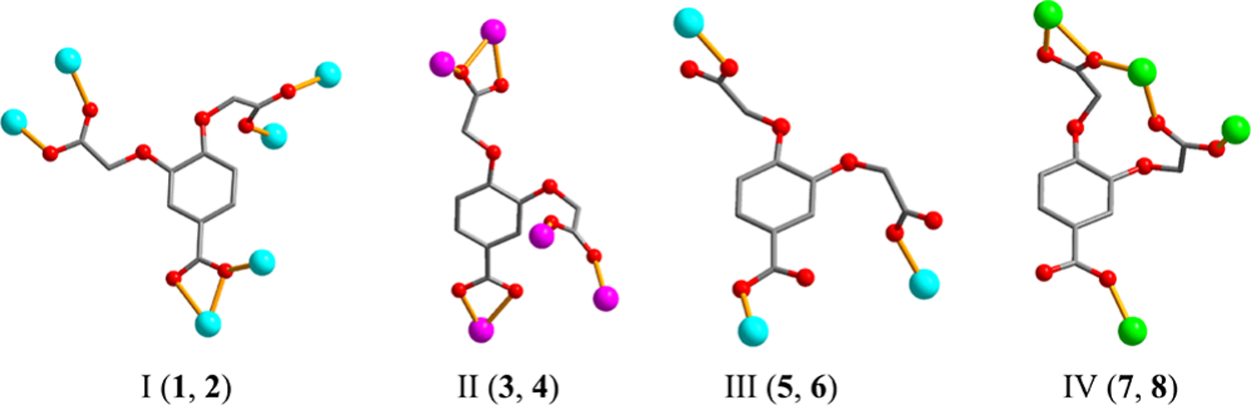
Coordination Modes of the cpbda^3–^ Linkers in Compounds **1**–**8**

### Structural Features

#### [M_3_(μ_6_-cpbda)_2_(phen)_2_]_*n*_·4nH_2_O (M = Zn­(**1**), Cd (**2**))

The 2D coordination polymers **1** and **2** are isostructural, and, therefore, only **1** is discussed herein (Figure 1). In the asymmetric unit of **1**, there are two zinc­(II) centers (Zn1 with 50% occupancy and Zn2 with 100% occupancy), a μ_6_-cpbda^3–^ block, a terminal phen ligand, and two crystallization H_2_O molecules. The Zn1 atom reveals a distorted octahedral {ZnO_6_} geometry, filled by six carboxylate oxygen atoms from six μ_6_-cpbda^3–^ units (Figure 1a). The Zn2 center is also six-coordinated and features a distorted octahedral {ZnN_2_O_4_} environment, which is occupied by four O atoms from three μ_6_-cpbda^3–^ linkers and two N_phen_ donors (Figure 1a). The cpbda^3–^ linkers exhibit a μ_6_-coordination mode (mode I, Scheme 2). One Zn1 and two Zn2 centers are held together via six carboxylate groups from six μ_6_-cpbda^3–^ linkers to form a Zn_3_ subunit (Figure 1b). These Zn_3_ subunits are further assembled, via the remaining carboxylate groups of μ_6_-cpbda^3–^ linkers, into an intricate 2D layer structure (Figure 1c). It can be defined as a trinodal 3,6-connected net with a 3,6,6L3 topology and point symbol of (4^12^.6^3^)­(4^3^)_2_(4^6^.6^9^)_2_ (Figure 1d).

**Figure 1:**
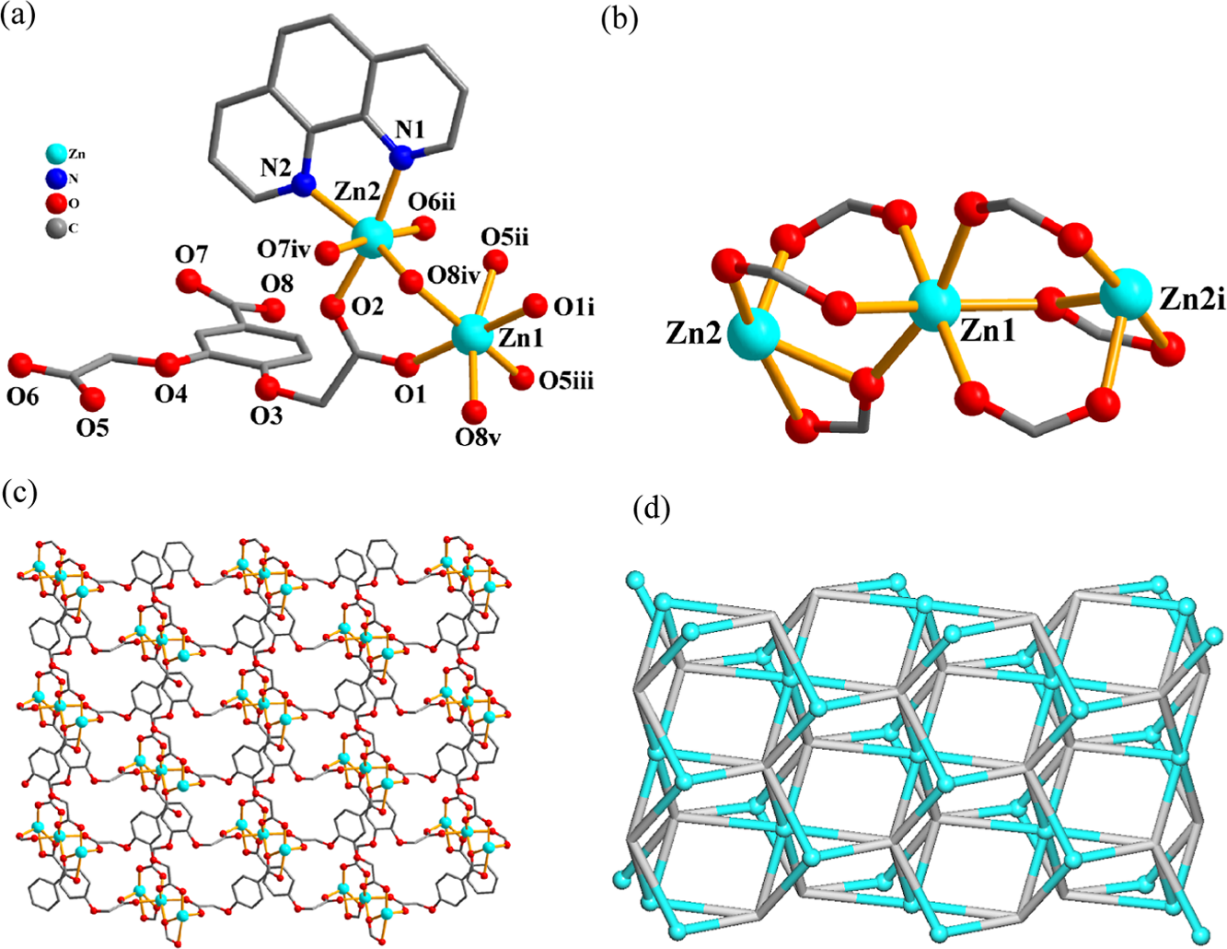
Structure of Zn CP **1**. (a) Coordination environment around the Zn­(II) atoms. (b) Zn_3_ subunit. (c) 2D layer seen along the *c* axis, and (d) its topological representation showing a 3,6,6L3 network (Zn, cyan balls; centroids of μ_6_-cpbda^3–^, gray).

#### [Co_3_(μ_5_-cpbda)_2_(μ-bipy)_2_]_*n*_·2*n*H_2_O (**3**) and [Zn_3_(μ_5_-cpbda)_2_(μ-bipy)_2_]_*n*_ (**4**)

These 3D metal–organic frameworks are isostructural, and only **3** is discussed below (Figure 2). Within an asymmetric unit, the structure of **3** possesses two Co­(II) atoms (Co1 with 50% occupancy and Co2 with 100% occupancy), a μ_5_-cpbda^3–^ linker, a bipy ligand, and one crystallization water molecule. The Co1 atom is 6-coordinated, forming a deformed octahedral {CoNO_5_} geometry, which is populated by five O donors from three μ_5_-cpbda^3–^ blocks and one N_bipy_ atom (Figure 2a). The Co2 center is also 6-coordinated and has an ideal octahedral {CoN_2_O_4_} environment, which is occupied by four O atoms from four μ_5_-cpbda^3–^ linkers and two N_bipy_ donors. The cpbda^3–^ block acts as a μ_5_-linker (type II, Scheme 2). One Co1 and two Co2 are assembled through four carboxylate groups of μ_5_-cpbda^3–^ into a Co3 subunit (Figure 2b). These are further interlinked, via the remaining carboxylate groups of μ_5_-cpbda^3–^ and additional μ-bipy linkers, into a 3D metal–organic framework (Figure 2c). From the topological perspective, this trinodal 4,5,6-linked framework (Figure 2d) is composed of the 4- and 6-connected Co2 and Co1 nodes, 5-connected μ_5_-cpbda^3–^ nodes, and μ-bipy linkers, resulting in a 4,5,6T22 topological net with the (4.6^5^)_2_(4^2^.6^6^.7.8)_2_(4^2^.6^8^.7^3^.8^2^) point symbol.

**Figure 2:**
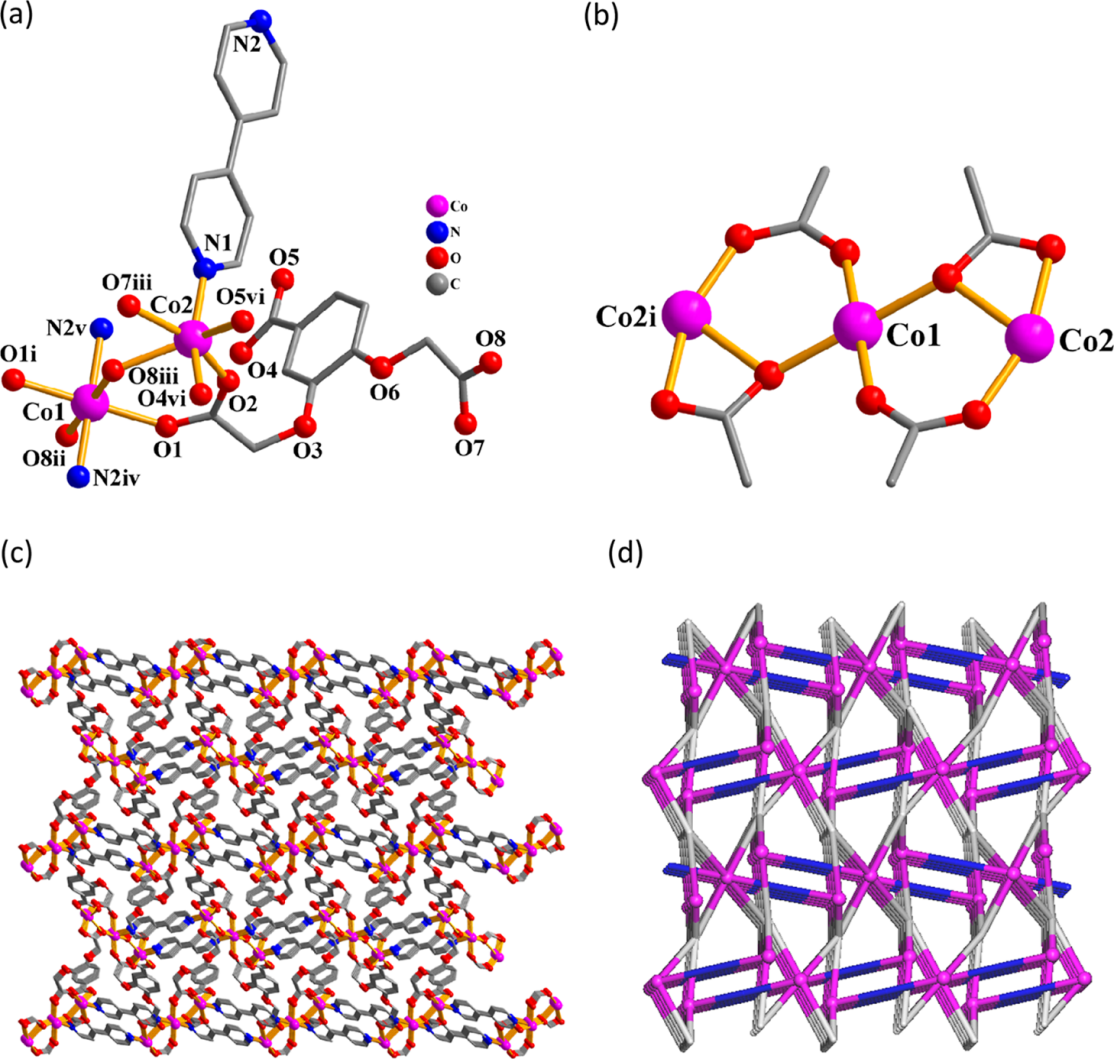
Structure of Co MOF **3**. (a) Coordination environment around the Co­(II) atoms. (b) Co_3_ subunit. (c) 3D MOF structure seen along the *a* axis, and (d) its topological representation showing a 4,5,6T22 framework (Co, magenta balls; centroids of μ_5_-cpbda^3–^, gray; centroids of μ-bipy, blue).

#### [Zn­(μ_3_-cpbda)­(Hbpa)]_*n*_·4nH_2_O (**5**)

The structure of this 2D coordination polymer is composed of one zinc­(II) atom, a μ_3_-cpbda^3–^ linker, a protonated Hbpa^+^ ligand, and four crystallization water molecules per asymmetric unit (Figure 3a). The Zn1 atom adopts a distorted tetrahedral {ZnNO_3_} geometry filled by three carboxylate O atoms from three μ_3_-cpbda^3–^ ligands and one N donor from the terminal Hbpa^+^ ligand. The cpbda^3–^ linkers display μ_3_-coordination (mode III, Scheme 2) and assemble the Zn1 atoms into a 2D layer (Figure 3b). This can be classified as n uninodal 3-linked net (Figure 3c) with a fes topology and a point symbol of (4.8^2^).

**Figure 3:**
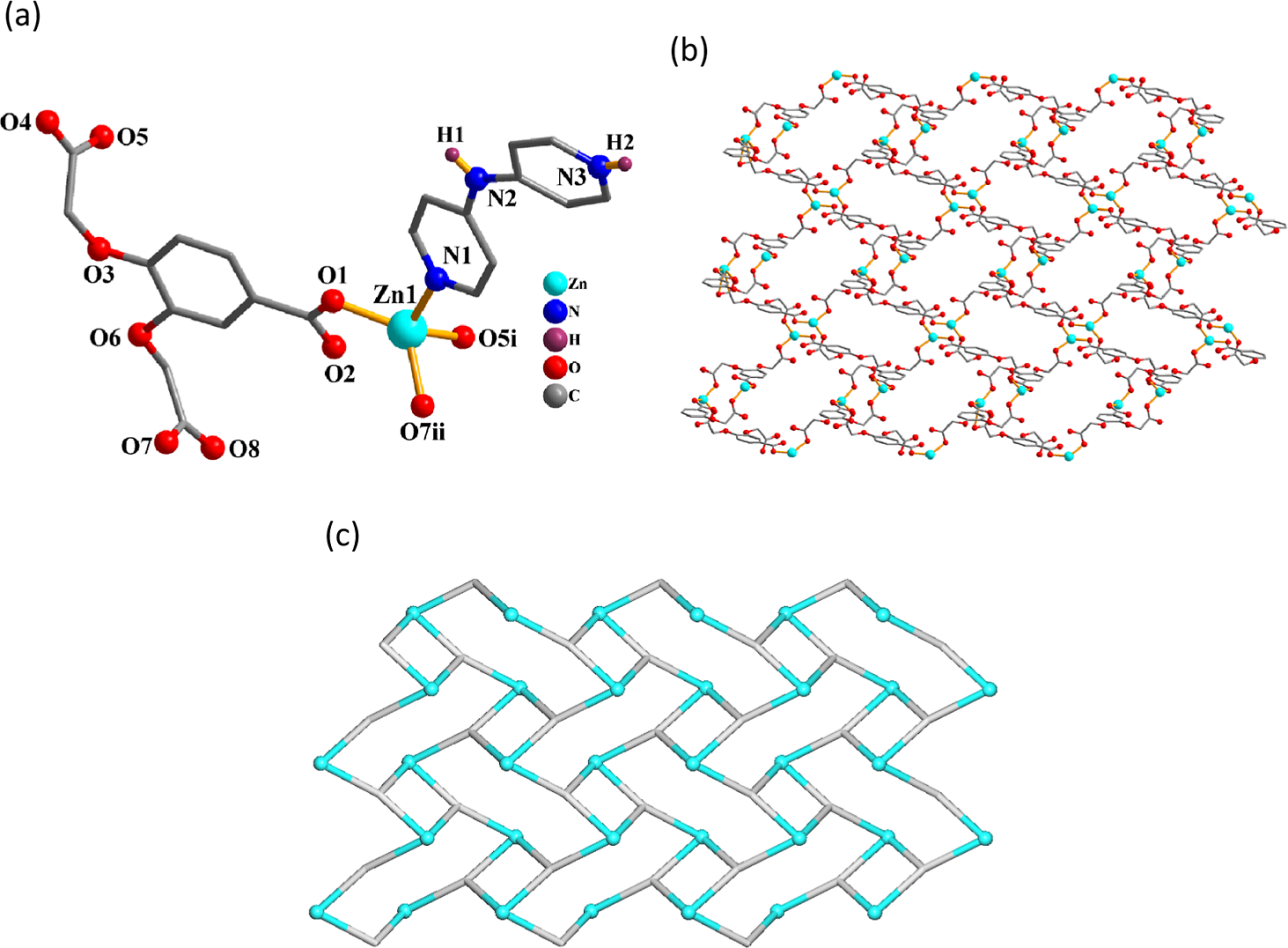
Structure of Zn CP **5**. (a) Coordination environment around the Zn­(II) atom. (b) 2D layer seen along the *c* axis (Hbpa^+^ ligands were omitted for clarity), and (c) its topological representation showing a fes network (Zn, cyan balls; centroids of μ_5_-cpbda^3–^, gray).

#### [Zn_4_(μ_3_-cpbda)_2_(μ-OH)_2_(μ-dpey)_3_(H_2_O)_2_]_*n*_·2*n*H_2_O (**6**)

In an asymmetric unit of this 2D coordination polymer (Figure 4), there are four Zn­(II) centers (Zn1–Zn4), two μ_3_-cpbda^3–^ blocks, two μ-OH^–^ groups, three μ-dpey linkers, and two coordinated and two lattice water molecules. The Zn1 and Zn3 centers are 5-coordinated and adopt distorted trigonal bipyramidal {ZnN_2_O_3_} geometries, which are populated by two N atoms from two μ-dpey linkers, one carboxylate O donor from μ_3_-cpbda^3–^, one μ-OH^–^ group, and one terminal H_2_O ligand (Figure 4a). The Zn2 and Zn4 centers are four-coordinated and reveal deformed tetrahedral {ZnNO_3_} environments. These are composed of two carboxylate oxygen atoms from two μ_3_-cpbda^3–^ linkers, one μ-OH^–^ group, and one N donor from μ-dpey. The cpbda^3–^ blocks function as μ_3_-linkers (mode III, Scheme 2) and assemble the {Zn_2_(μ-OH)}^3+^ units into 1D ladder-like motifs. These motifs are held together by μ-dpey linkers to form a 2D layer (Figure 4b). Topologically, the resulting layer can be defined as a binodal 3,6-linked net composed of the 6-connected {Zn_2_(μ-OH)}^3+^ and the 3-connected μ_3_-cpbda^3–^ nodes, as well as the μ-dpey linkers (Figure 4c). This layer has a 3,6L77 topology and a point symbol of (4^2^.6)­(4.^8^6^7^).

**Figure 4:**
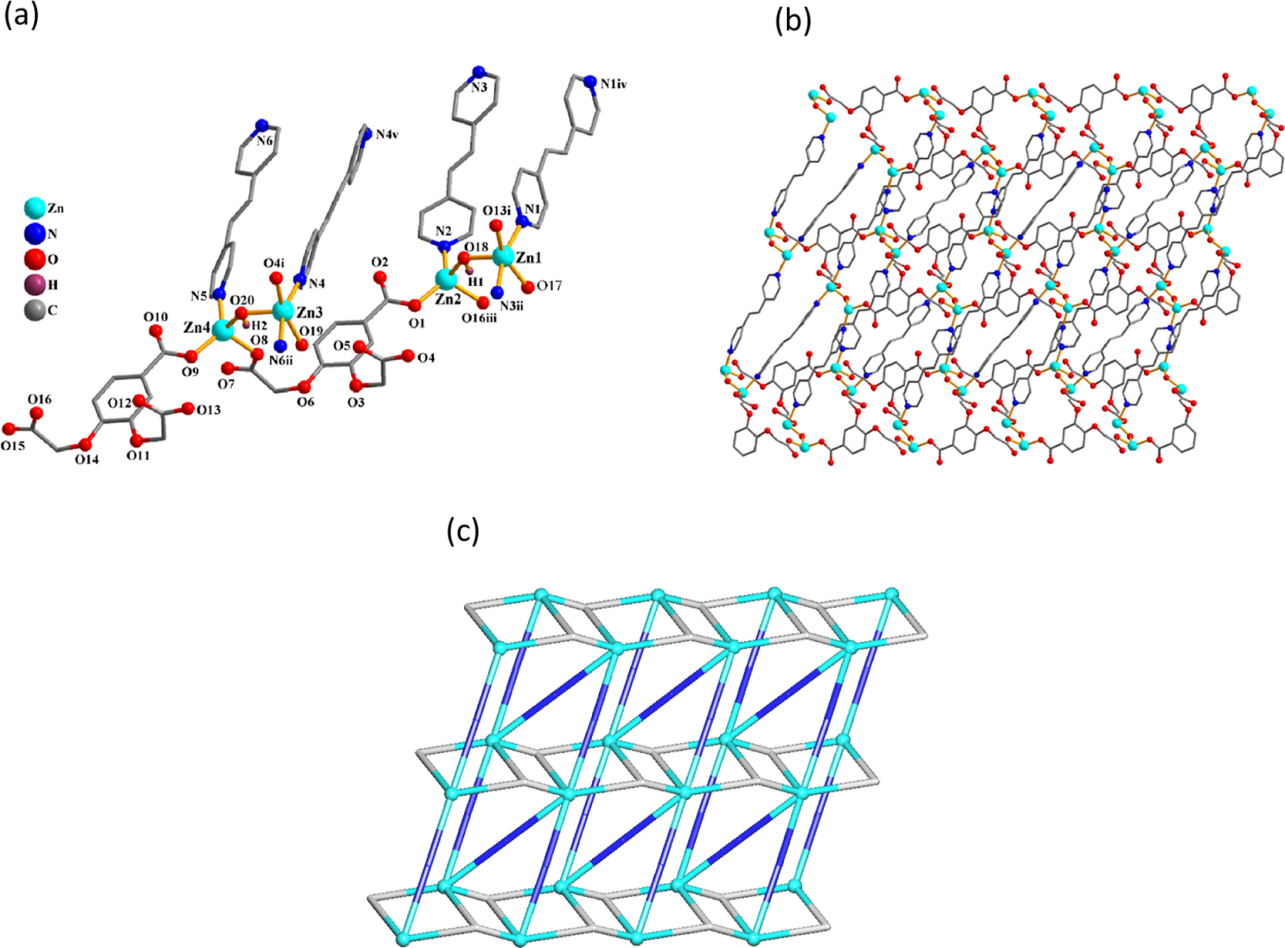
Structure of Zn CP **6**. (a) Coordination environment around the Zn­(II) atoms. (b) 2D layer seen along the *a* axis, and (c) its simplified topological representation showing a 3,6L77 network (centroids of {Zn_2_(μ-OH)}^3+^ units, cyan balls; centroids of μ_3_-cpbda^3–^, gray; centroids of μ-dpey, blue).

#### [Co_3_(μ_4_-cpbda)_2_(μ-dpey)_3_]_*n*_·2*n*H_2_O (**7**) and [Ni_3_(μ_4_-cpbda)_2_(μ-dpea)_3_]_*n*_·2*n*H_2_O (**8**)

As these two 3D metal–organic frameworks feature similar structures, compound **8** is discussed as an example (Figure 5). The asymmetric unit of **8** contains two nickel­(II) centers (Ni1 with 100% occupancy and Ni2 with 50% occupancy), a μ_4_-cpbda^3–^ block, one and a half of μ-dpey linker, and two water molecules of crystallization. The Ni1 center is 6-coordinated and shows a distorted octahedral {NiN_2_O_4_} environment, which is populated by four carboxylate O atoms from four μ_4_-cpbda^3–^ ligands and two N atoms from two additional μ-dpey linkers (Figure 5a). The Ni2 center is also 6-coordinated and reveals an ideal octahedral {NiN_2_O_4_} coordination geometry, which is filled by four O atoms from two μ_4_-cpbda^3–^ linkers and two N donors from two μ-dpey ligands. The cpbda^3–^ linkers exhibit μ_4_-coordination (mode IV, Scheme 2). The trimeric Ni3 subunits are formed by bridging two Ni1 and one Ni2 centers through four carboxylate groups from two μ_4_-cpbda^3–^ ligands (Figure 5b). These Ni3 subunits are linked by the μ_4_-cpbda^3–^ blocks into 2D layer motifs, which are further extended by μ-dpey pillars into an intricate 3D metal–organic framework (Figure 5c). Topologically, this framework is assembled from the 4- and 5-connected Ni2 and Ni1 centers, 4-connected μ_4_-cpbda^3–^ blocks, and 2-connected μ-dpey pillars (Figure 5d). As a result, a trinodal 4,4,5-linked net is generated that features a unique topology with a point symbol of (4.6^4^.8)­(4.6^6^.8^3^)_2_(4^3^.6^3^)_2_.

**Figure 5:**
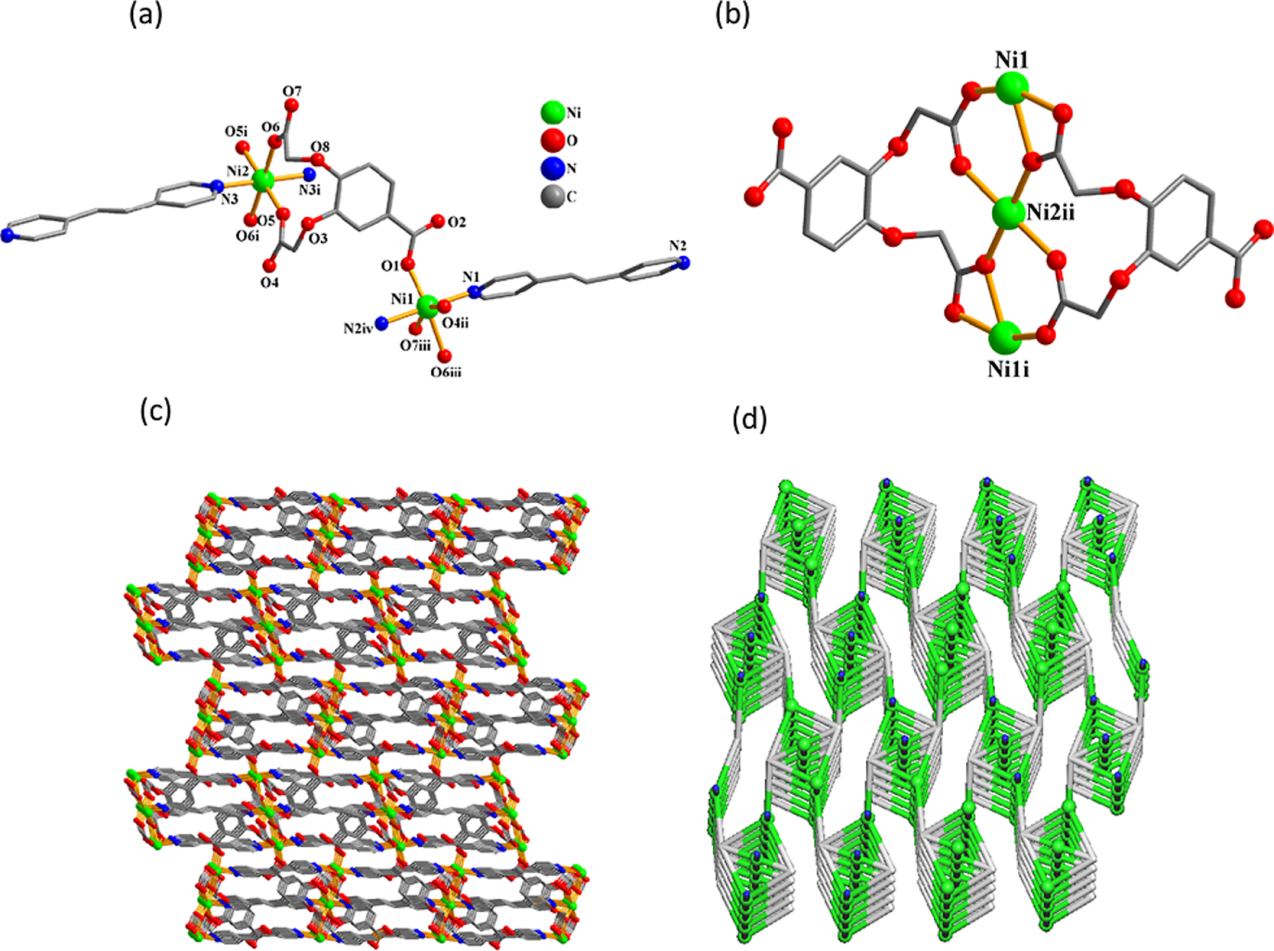
Structure of Ni MOF **8**. (a) Coordination environment around the Ni­(II) atoms. (b) Ni_3_ unit. (c) 3D framework seen along the *a* axis, and (d) its topological representation showing a topologically unique net (view along the *c* axis; Ni, green balls; centroids of μ_4_-cpbda^3–^, gray; centroids of μ-dpey, blue).

### Thermal Analysis

Thermogravimetric analyses (TGA) of **1**–**8** were conducted under an inert nitrogen atmosphere over the temperature range of 25–800 °C (Figure 6). CP **1** reveals a mass loss between 60 and 174 °C due to the release of four crystallization water molecules (observed 6.0%; calculated 6.2%); the decomposition of the dehydrated sample starts at 311 °C. Similarly, a release of four lattice water molecules (observed 5.7%; calculated 5.5%) from CP **2** occurs between 79 and 132 °C, followed by the decomposition at 327 °C. For MOF **3**, a mass decrease (observed 3.1%; calculated 3.4%) in the 72–131 °C interval is due to the removal of two crystallization H_2_O molecules; the dehydrated sample remains stable up to 392 °C. Compound **4** has no coordinated or crystallization water molecules, and its metal–organic framework remains stable on heating up to 378 °C. CP **5** shows a mass loss between 30 and 97 °C, attributable to a release of four lattice water molecules (observed 12.4%; calculated 12.5%); the decomposition of a dehydrated sample begins at 275 °C. CP **6** undergoes disintegration starting at 236 °C, preceded by a release of two lattice and two coordinated water molecules in the 35–128 °C interval (observed 4.8%; calculated 5.0%). For MOF **7**, a mass decrease (observed 3.0%; calculated 2.8%) between 57 and 156 °C is due to the removal of two crystallization H_2_O molecules, followed by the decomposition starting above 336 °C. The TGA of MOF **8** also shows a weight loss between 90 and 218 °C, associated with the release of two crystallization water molecules (observed 2.6%; calculated 2.8%); the dehydrated framework remains stable up to 353 °C.

**Figure 6:**
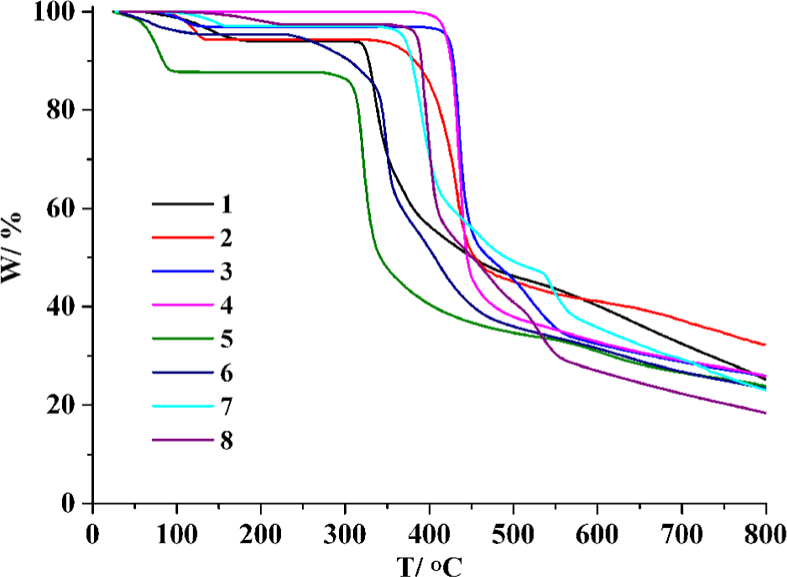
TGA curves for compounds **1**–**8**.

### Catalytic Properties

Coordination polymers are known as catalysts for various condensation reactions.^23,28,29,32^ In this study, we explored the obtained CPs **1**–**8** as heterogeneous catalysts in the condensation reaction between an aldehyde and a nitrile derivative. As model substrates, benzaldehyde and malononitrile were used. The reaction was carried out at 25 °C in methanol as a typical solvent, leading to the selective generation of 2-benzylidenemalononitrile (Scheme 3 and Table 3). Additionally, the effects of different parameters, including catalyst loading and recyclability, reaction time, solvent type, and substrate scope, were investigated. In particular, the substrate scope included benzaldehydes with different substituents as well as 1-naphthaldehyde and 9-anthraldehyde. These were tested in coupling reactions with malononitrile and ethyl cyanoacetate.

**Scheme 3:**
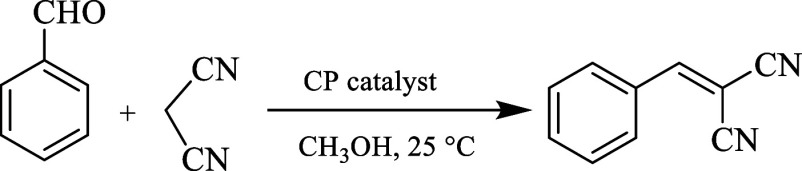
CP-Catalyzed Condensation of Benzaldehyde with Malononitrile

**Table 3:** CP-Catalyzed Condensation of Benzaldehyde with Malononitrile^a^

entry	catalyst	reaction time, min	catalyst loading, mol %	solvent	product yield,%^b^
1	**5**	10	2.0	CH_3_OH	46
2	**5**	20	2.0	CH_3_OH	61
3	**5**	30	2.0	CH_3_OH	75
4	**5**	40	2.0	CH_3_OH	87
5	**5**	50	2.0	CH_3_OH	95
6	**5**	60	2.0	CH_3_OH	99
7	**5**	60	2.0	H_2_O	96
8	**5**	60	2.0	C_2_H_5_OH	96
9	**5**	60	2.0	CH_3_CN	88
10	**5**	60	2.0	CHCl_3_	66
11	**5**	60	1.0	CH_3_OH	95
12	**1**	60	2.0	CH_3_OH	88
13	**2**	60	2.0	CH_3_OH	87
14	**3**	60	2.0	CH_3_OH	84
15	**4**	60	2.0	CH_3_OH	82
16	**6**	60	2.0	CH_3_OH	99
17	**7**	60	2.0	CH_3_OH	82
18	**8**	60	2.0	CH_3_OH	81
19	blank	60	–	CH_3_OH	16
20	ZnCl_2_	60	2.0	CH_3_OH	27
21	H_3_cpbda	60	2.0	CH_3_OH	28

a Conditions: benzaldehyde (0.5 mmol), malononitrile (1.0 mmol), catalyst (1–2 mol %), solvent (1.0 mL), 25 °C.

b Yield based on ^1^H NMR analysis: [moles of product per mol of aldehyde substrate] × 100%.

Among the obtained compounds, the Zn-based CPs **5** and **6** revealed the highest catalytic activity, converting benzaldehyde to 2-benzylidenemalononitrile with the yields as high as 99% (Table 3 and Figures S3 and S5). Although there is no particular difference in the catalytic performance of **5** and **6**, compound **5** contains only 4-coordinated Zn­(II) centers (vs 4- and 5-coordinated Zn­(II) atoms in **6**) and was thus selected as a representative example to evaluate the influence of various reaction parameters. For example, when the reaction time is extended from 10 to 60 min (entries 1–6 in Table 3), the product yield increases from 46 to 99%. The increase in the catalyst loading from 1 to 2 mol % also boosts the product yield from 95 to 99% (entries 6 and 11 in Table 3). We also screened alternative solvents, such as water, ethanol, acetonitrile, and chloroform, but these led to lower product yields than those in methanol, namely, ranging from 66 to 96%. It should be mentioned that very high product yields (96%, entries 7 and 8, Table 3) can be obtained in water or ethanol that are considered as green solvents. Control tests showed that the condensation reaction is much less pronounced when using H_3_cpbda (28% yield) or ZnCl_2_ (27% yield) as catalysts (entries 19–21, Table 3). In the absence of a catalyst (blank test), the yield decreased to 16%.

In contrast to CPs **5** and **6**, other obtained coordination polymers revealed lower activity, with maximum yields between 81 and 88% (entries 12–18, Table 3). This difference in the catalytic activity might be attributed to the presence of Zn­(II) centers with unsaturated coordination sites in **5** and **6** and a better accessibility of active sites in 2D coordination polymers.^23,32^ In fact, Zn-based coordination polymers often outperform Cd, Ni, and Co analogues in these catalytic reactions, because Zn­(II) is a borderline Lewis acid with fast ligand exchange kinetics, enabling a more efficient activation of the carbonyl group. In addition, a closed d^10^ configuration of Zn^2+^ may also prevent redox-induced deactivation pathways that can occur in other metal centers. Additionally, Zn­(II) tends to form more labile, less strongly chelating metal-linker bonds, generating coordinatively unsaturated sites and surface basicity that promote aldehyde activation and nitrile-stabilized carbanion formation.^28,29^ It should also be mentioned that the new coordination compounds reported in the present study are not porous, and the catalysis is external surface-based.

A number of substituted benzaldehyde substrates was tested to explore the substrate scope in the condensation reaction with malononitrile. These studies were performed under optimized conditions (1 h reaction time, 25 °C, 2.0 mol %, catalyst **5**, methanol solvent). The corresponding products were obtained in yields ranging from 56 to 99% (Table S3). Benzaldehyde substrates containing strong electron-withdrawing groups, such as nitro (–NO_2_) and chloro (–Cl), showed the highest reactivity, likely due to the increased electrophilicity of the substrates (entries 2–5, Table S3). In contrast, benzaldehyde substrates with electron-donating groups, such as methyl (–CH_3_) or methoxy (–OCH_3_), resulted in lower product yields (entries 6 and 7, Table S3).

In addition, the recyclability of catalyst **5** was investigated by performing several reaction cycles with the same sample of the catalyst. After each cycle of the reaction between benzaldehyde and malononitrile, the catalyst was recovered by centrifugation, washed with methanol, air-dried at 25 °C, and then used again. The obtained results show that CP **5** maintains its original activity for at least five cycles (Figure S6), leading to product yields of 99%, 99%, 97%, and 95% in the second through fifth runs, respectively. Furthermore, PXRD patterns of parent CP **5** and the reused catalyst confirmed that its structure is preserved (Figure S7), despite observing some novel signals and peak broadening. These changes, which are expectable in the catalyst recycling experiments, likely result from impurities or a decrease in crystallinity. The catalytic performance of CPs **5** and **6** in this type of model condensation reactions is comparable to or even superior than that of other heterogeneous catalysts based on metal-carboxylate CPs (Table S4).^44–48^ In particular, catalysts **5** and **6** feature higher turnover numbers and turnover frequencies (up to 50 h^–1^), leading to almost quantitative product yields obtained within a shorter reaction time (1 h) under room temperature conditions.

To further explore the scope of the present reaction to a different nitrile derivative, compounds **1**–**8** were investigated in the condensation of benzaldehyde with ethyl cyanoacetate to give ethyl-2-cyano-3-phenyl acrylate (Scheme 4). As in the case of the coupling reaction between benzaldehyde and malononitrile (Table 3), the effects of different reaction parameters were studied in the system with ethyl cyanoacetate, including the effects of reaction time (Figure S9), catalyst loading, and solvent type (Table S5), as well as catalyst recycling (Figure S10). In addition, different aldehyde substrates were screened in the reaction with ethyl cyanoacetate (Table S6 and Figure S4). The obtained results essentially resemble those in the system with malononitrile, although the condensation reactions between aldehydes and ethyl cyanoacetate require longer reaction times (up to 4 h) and a slightly increased temperature (40 °C). The best product yields (up to 99%) were also attained in the presence of Zn-based catalysts **5** and **6**.

**Scheme 4:**
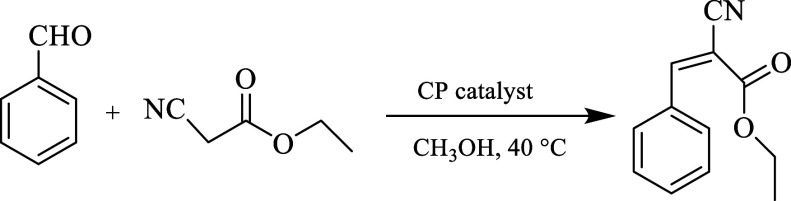
CP-Catalyzed Condensation of Benzaldehyde with Ethyl Cyanoacetate

## Conclusions

In the present research study, we applied a hydrothermal method to assemble eight new coordination polymers and metal–organic frameworks, using a still little explored 2,2′-((4-carboxy-1,2-phenylene)*bis*(oxy))­diacetic acid (H_3_cpbda) as the primary linker. The obtained compounds were fully characterized, and their crystal structures were determined by single-crystal X-ray diffraction. The products revealed different types of 2D layer networks (**1**, **2**, **5**, and **6**) or 3D frameworks (**3**, **4**, **7**, and **8**) with distinct topologies. The diversity in coordination modes of the cpbda^3–^ linkers is likely influenced by the nature of the metal­(II) centers and the *N*,*N*-donor auxiliary ligands. In addition, all of the obtained products were screened as heterogeneous catalysts in the model condensation reactions between benzaldehyde and malononitrile (or ethyl cyanoacetate). Remarkably, the Zn-based coordination polymer **5** showed high catalytic activity (up to 99% product yields) and reusability (up to 5 catalytic cycles). Under optimized conditions, the substrate scope of the condensation reactions was also extended to other aldehyde substrates.

In summary, this work widens a limited family of coordination polymers derived from H_3_cpbda, illustrating the versatility of this flexible tricarboxylic acid as a linker for the design of new CPs and MOFs. We anticipate that future studies will further investigate the use of H_3_cpbda as a linker for designing new metal–organic architectures with varied functional properties and promising applications in heterogeneous catalysis and beyond.

## Supplementary Material



## Notes

Materials and methods, synthesis and analytical data for compounds **1**–**8**, FTIR spectra, PXRD patterns, additional catalysis data, and bonding parameters (PDF)
