# Supplementary material for: Coordination Polymers Assembled from Flexible Tricarboxylate Linkers: Hydrothermal Synthesis, Structural Diversity, and Catalytic Features
Source: Inorg Chem. 2026 Feb 7;65(7):3967–76. doi: 10.1021/acs.inorgchem.5c05274 (PMC12933886; doi:10.1021/acs.inorgchem.5c05274)
Supplement: Supplementary file 1 [file ic5c05274_si_001.pdf]

Supporting Information

Coordination Polymers Assembled from Flexible Tricarboxylate Linkers: Hydrothermal Synthesis, Structural Diversity, and Catalytic Features

Wei Dou,[#,*] Beining Shi,[#] Xiaoxiang Fan,[§] Jinzhong Gu,[#,*] Marina V. Kirillova,[†] and Alexander M. Kirillov[†,*]

[#]*State Key Laboratory of Natural Product Chemistry, College of Chemistry and Chemical Engineering, Lanzhou University, Lanzhou 730000, People's Republic of China*

[§]*Nuclear Power Institute of China, Chengdu 610041, People's Republic of China*

[†]*MINDlab: Molecular Design & Innovation Laboratory, Centro de Química Estrutural, Institute of Molecular Sciences, Departamento de Engenharia Química, Instituto Superior Técnico, Universidade de Lisboa, Av. Rovisco Pais, 1049-001, Lisbon, Portugal*

*Corresponding authors, E-mail: douwei@lzu.edu.cn (W. Dou), gujzh@lzu.edu.cn (J.-Z. Gu), kirillov@tecnico.ulisboa.pt (A. M. Kirillov).

Supporting Information contains: materials and methods, synthesis and analytical data for compounds **1**–**8**, FTIR spectra (Figure S1), PXRD patterns (Figure S2), additional catalysis data (Figures S3–S9, Tables S3–S6), bonding parameters (Tables S1 and S2), and crystallographic data in CIF format (CCDC-2500238–2500245). (PDF).

Materials and Methods

All chemicals and solvents were obtained from commercial suppliers. 2,2'-((4-Carboxy-1,2-phenylene)bis(oxy))diacetic acid (H_3cpbda) was acquired from Yanshen Technology Co., Ltd. C/N/H analyses were run on an Elementar Vario EL elemental analyzer. Bruker EQUINOX 55 spectrometer was used for recording the FTIR spectra (KBr discs). LINSEIS STA PT1600 thermal analyzer was used for thermogravimetric (TGA) measurements (heating rate: 10°C/min; N_2 flow). PXRD (powder X-ray diffraction) analyses were carried out on a Rigaku-Dmax 2400 diffractometer (Cu-Kα radiation, λ = 1.54060 Å). Solution ^{1}H NMR spectra were recorded on a JNM ECS 400 M spectrometer.

Synthesis and analytical data for 1–8

Synthesis of [Zn_3(μ_6-cpbda)$_2$(phen)$_2$]$_n$·4$n$$H_2O$ (1). A mixture of $ZnCl_2$ (40.9 mg, 0.30 mmol), H_3cpbda (54.0 mg, 0.20 mmol), phen (59.4 mg, 0.30 mmol), NaOH (24.0 mg, 0.60 mmol), and H_2O (10 mL) was stirred at room temperature for 15 min, then sealed in a 25 mL Teflon-lined stainless steel vessel, and heated at 160 °C for 3 days, followed by cooling to room temperature at a rate of 10 °C·h^{-1}. Colorless block-shaped crystals of **1** were isolated manually, washed with distilled water and dried (yield: 43% based on H_3cpbda). Anal. Calcd for $C_{46}H_{38}Zn_3N_4O_{20}$: C, 47.50; H, 3.29; N, 4.82. Found: C, 47.71; H, 3.31; N, 4.85%. FTIR (KBr, cm^{-1}): 3591 w, 3510 w, 3072 w, 1644 m, 1613 s, 1513 m, 1448 s, 1428 s, 1378 w, 1336 w, 1274 m, 1217 m, 1156 w, 1128 w, 1057 w, 959 w, 924 w, 882 w, 851 w, 824 w, 774 m, 724 m, 644 m, 617 w, 590 w.

Synthesis of [Cd_3(μ_6-cpbda)$_2$(phen)$_2$]$_n$·4$n$$H_2O$ (2). A mixture of $CdCl_2 \cdot H_2O$ (60.3 mg, 0.30 mmol), H_3cpbda (54.0 mg, 0.20 mmol), phen (59.4 mg, 0.30 mmol), NaOH (24.0 mg, 0.60 mmol), and H_2O (10 mL) was stirred at room temperature for 15 min, then sealed in a 25 mL Teflon-lined stainless steel vessel, and heated at 160 °C for 3 days, followed by cooling to room temperature at a rate of 10 °C·h^{-1}. Colorless block-shaped crystals of **2** were isolated manually, washed with distilled water and dried (yield: 45% based on H_3cpbda). Anal. Calcd for $C_{46}H_{38}Cd_3N_4O_{20}$: C, 42.37; H, 2.94; N, 4.30. Found: C, 42.15; H, 2.96; N, 4.14%. FTIR (KBr, cm^{-1}): 3586 w, 3464 w, 3072 w, 2921 w, 1605 s, 1513 m, 1421 s, 1382

w, 1340 w, 1267 m, 1217 m, 1125 w, 1052 w, 963 w, 921 w, 882 w, 851 w, 825 m, 774 m, 724 m, 644 m, 613 w, 586 w.

Synthesis of [Co_3(μ_5-cpbda)$_2$(μ-bipy)$_2$]$_n$·2$n$$H_2O$ (3). A mixture of $CoCl_2·6H_2O$ (71.4 mg, 0.30 mmol), H_3cpbda (54.0 mg, 0.20 mmol), 4,4′-bipyridine (46.8 mg, 0.30 mmol), NaOH (24.0 mg, 0.60 mmol), and H_2O (10 mL) was stirred at room temperature for 15 min, then sealed in a 25 mL Teflon-lined stainless steel vessel, and heated at 160 °C for 3 days, followed by cooling to room temperature at a rate of 10 °C·h^{-1}. Pink block-shaped crystals of **3** were isolated manually, washed with distilled water and dried (yield: 43% based on H_3cpbda). Anal. Calcd for $C_{42}H_{34}Co_3N_4O_{18}$: C, 47.61; H, 3.23; N, 5.29. Found: C, 47.36; H, 3.21; N, 5.40%. FTIR (KBr, cm^{-1}): 3598 w, 3487 w, 3075 w, 2922 w, 2852 w, 1609 s, 1537 w, 1510 w, 1433 s, 1378 w, 1336 w, 1271 m, 1213 m, 1144 w, 1113 m, 1067 w, 1051 w, 959 w, 936 w, 878 w, 809 m, 779 m, 725 m, 632 m, 613 w, 594 w.

Synthesis of [Zn_3(μ_5-cpbda)$_2$(μ-bipy)$_2$]$_n$ (4). A mixture of $ZnCl_2$ (40.9 mg, 0.30 mmol), H_3cpbda (54.0 mmol, 0.20 mmol), 4,4′-bipyridine (46.8 mg, 0.30 mmol), NaOH (24.0 mg, 0.60 mmol), and H_2O (10 mL) was stirred at room temperature for 15 min, then sealed in a 25 mL Teflon-lined stainless steel vessel, and heated at 160 °C for 3 days, followed by cooling to room temperature at a rate of 10 °C·h^{-1}. Colorless block-shaped crystals of **4** were isolated manually, washed with distilled water and dried (yield: 46% based on H_3cpbda). Anal. Calcd for $C_{42}H_{30}Zn_3N_4O_{16}$: C, 48.37; H, 2.90; N, 5.37. Found: C, 48.74; H, 2.87; N, 5.40%. FTIR (KBr, cm^{-1}): 3598 w, 3479 w, 3075 w, 2960 w, 1613 s, 1544 m, 1424 s, 1378 m, 1340 w, 1267 s, 1213 m, 1113 w, 1058 m, 932 w, 878 w, 809 m, 728 m, 636 m.

Synthesis of [Zn(μ_3-cpbda)(Hbpa)]$_n$·4$n$$H_2O$ (5). A mixture of $ZnCl_2$ (40.9 mg, 0.30 mmol), H_3cpbda (54.0, 0.20 mmol), bpa (51.3 mg, 0.30 mmol), NaOH (24.0 mg, 0.60 mmol), and H_2O (10 mL) was stirred at room temperature for 15 min, then sealed in a 25 mL Teflon-lined stainless steel vessel, and heated at 160 °C for 3 days, followed by cooling to room temperature at a rate of 10 °C·h^{-1}. Colorless block-shaped crystals of **5** were isolated manually, washed with distilled water and dried (yield: 42% based on H_3cpbda). Anal. Calcd for $C_{21}H_{25}ZnN_3O_{12}$: C, 43.72; H, 4.37; N, 7.28. Found: C, 43.45; H, 4.39; N, 7.25%. FTIR (KBr, cm^{-1}): 3525 m, 3414 m, 3302 w, 3091 w, 1717 w, 1609 s, 1513 s, 1425 m, 1352 m, 1217 w, 1210 m, 1125 w, 1055 w, 1025 w, 936 w, 820 m, 778 w, 721 w, 648 w, 597 w.

Synthesis of $[Zn_4(\mu_3\text{-cpbda})_2(\mu\text{-OH})_2(\mu\text{-dpey})_3(H_2O)_2]_n\cdot 2nH_2O$ (6). A mixture of $ZnCl_2$ (40.9 mg, 0.30 mmol), H_3cpbda (54.0, 0.20 mmol), dpey (54.6 mg, 0.30 mmol), NaOH (24.0 mg, 0.60 mmol), and H_2O (10 mL) was stirred at room temperature for 15 min, then sealed in a 25 mL Teflon-lined stainless steel vessel, and heated at 160 °C for 3 days, followed by cooling to room temperature at a rate of 10 °C·h^{-1}. Colorless block-shaped crystals of **6** were isolated manually, washed with distilled water and dried (yield: 33% based on H_3cpbda). Anal. Calcd for $C_{58}H_{54}Zn_4N_6O_{22}$: C, 48.09; H, 3.76; N, 5.80. Found: C, 48.33; H, 3.74; N, 5.83%. FTIR (KBr, cm^{-1}): 3419 w, 3142 w, 2923 w, 1617 s, 1575 m, 1499 w, 1424 m, 1361 s, 1265 w, 1202 w, 1186 w, 1134 w, 1105 w, 1050 w, 1016 w, 970 w, 924 w, 895 w, 831 m, 785 w, 752 w, 642 w, 551 m.

Synthesis of $[Co_3(\mu_4\text{-cpbda})_2(\mu\text{-dpey})_3]_n\cdot 2nH_2O$ (7). A mixture of $CoCl_2\cdot 6H_2O$ (71.4 mg, 0.30 mmol), H_3cpbda (54.0 mg, 0.20 mmol), dpey (54.6 mg, 0.30 mmol), NaOH (24.0 mg, 0.60 mmol), and H_2O (10 mL) was stirred at room temperature for 15 min, then sealed in a 25 mL Teflon-lined stainless steel vessel, and heated at 160 °C for 3 days, followed by cooling to room temperature at a rate of 10 °C·h^{-1}. Pink block-shaped crystals of **7** were isolated manually, washed with distilled water and dried (yield: 44% based on H_3cpbda). Anal. Calcd for $C_{58}H_{48}Co_3N_6O_{18}$: C, 53.84; H, 3.74; N, 6.50. Found: C, 53.54; H, 3.76; N, 6.53%. FTIR (KBr, cm^{-1}): 3629 w, 3453 w, 3066 w, 2936 w, 1613 s, 1567 m, 1495 w, 1445 w, 1419 w, 1356 s, 1289 w, 1265 w, 1218 w, 1185 w, 1101 w, 1046 w, 1016 w, 975 w, 899 w, 831 m, 785 w, 752 w, 701 w, 626 w, 584 w, 555 m.

Synthesis of $[Ni_3(\mu_4\text{-cpbda})_2(\mu\text{-dpea})_3]_n\cdot 2nH_2O$ (8). A mixture of $NiCl_2\cdot 6H_2O$ (71.3 mg, 0.30 mmol), H_3cpbda (54.0 mg, 0.20 mmol), dpea (55.2 mg, 0.30 mmol), NaOH (24.0 mg, 0.60 mmol), and H_2O (10 mL) was stirred at room temperature for 15 min, then sealed in a 25 mL Teflon-lined stainless steel vessel, and heated at 160 °C for 3 days, followed by cooling to room temperature at a rate of 10 °C·h^{-1}. Green block-shaped crystals of **8** were isolated manually, washed with distilled water and dried (yield: 47% based on H_3cpbda). Anal. Calcd for $C_{58}H_{54}Ni_3N_6O_{18}$: C, 53.62; H, 4.19; N, 6.47. Found: C, 53..27; H, 4.17; N, 6.51%. FTIR (KBr, cm^{-1}): 3419 w, 3032 w, 1612 s, 1567 m, 1491 w, 1445 w, 1424 w, 1398 w, 1361 s, 1294 w, 1260 w, 1218 w, 1185 w, 1105 w, 1041 w, 1016 w, 975 w, 895 w, 869 w, 831 m, 772 m, 752 w, 739 w, 698 w, 626 w, 551 m.

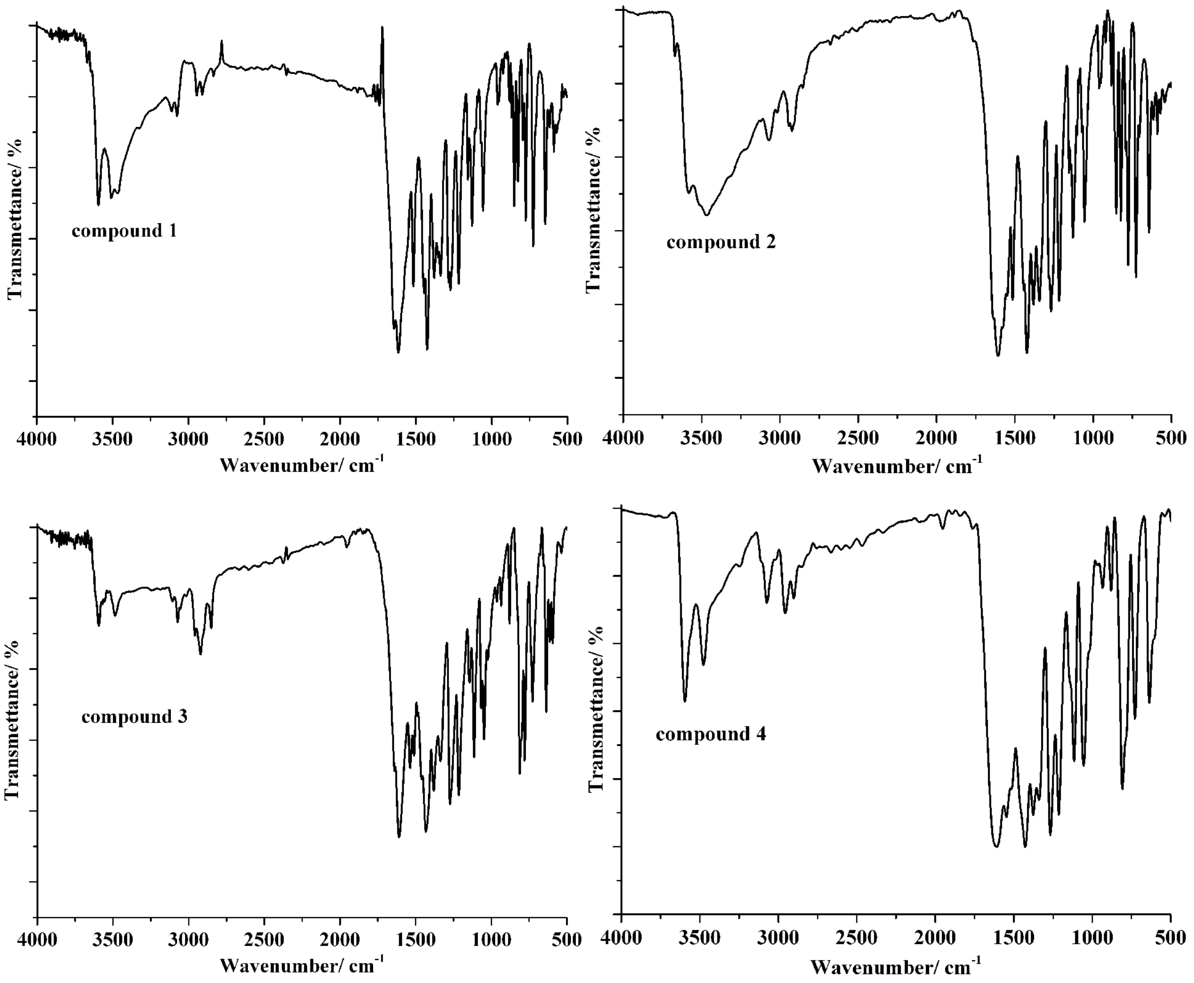

Figure S1a. FTIR spectra of compounds **1**–**4**.

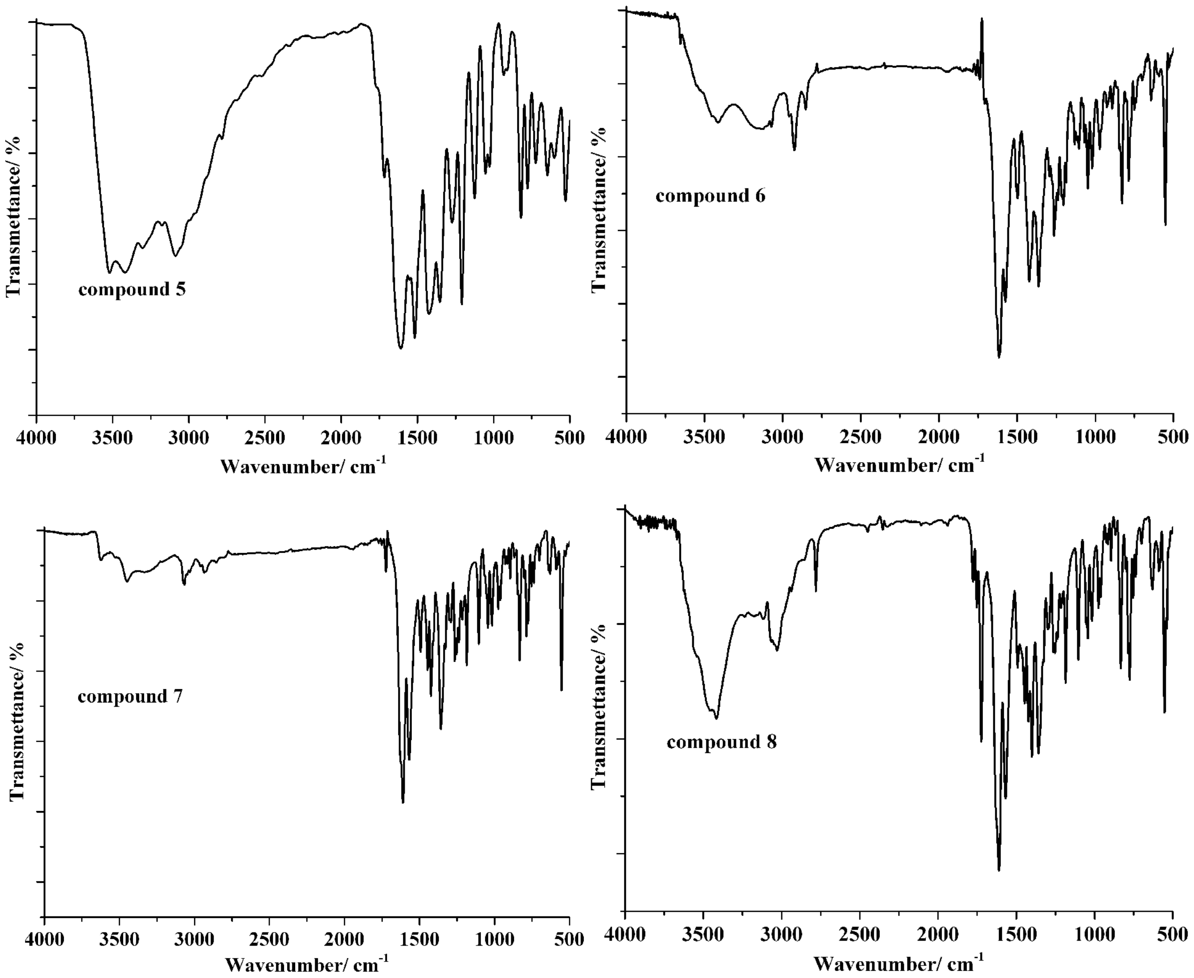

Figure S1b. FTIR spectra of compounds **5**–**8**.

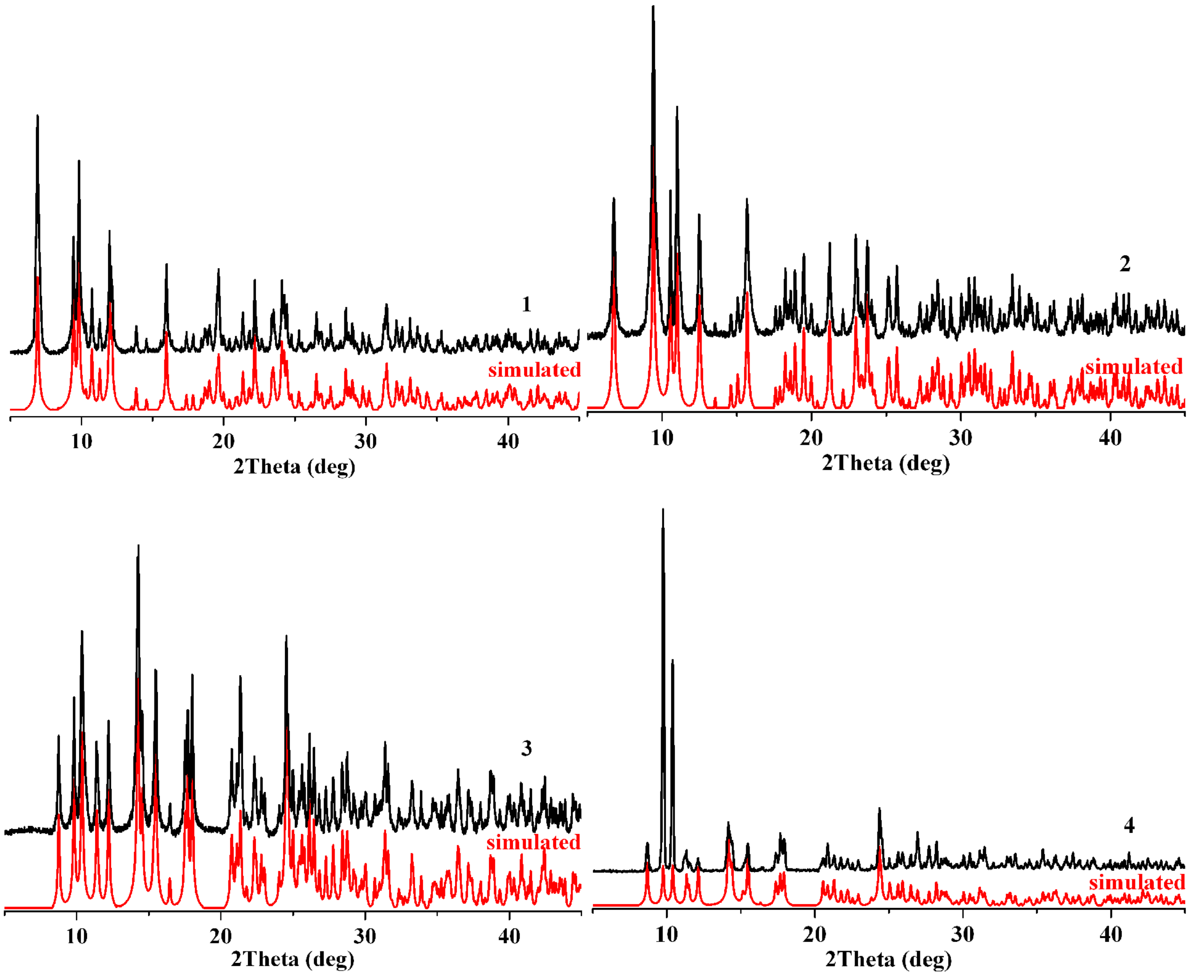

Figure S2a. PXRD patterns of compounds **1–4** at room temperature.

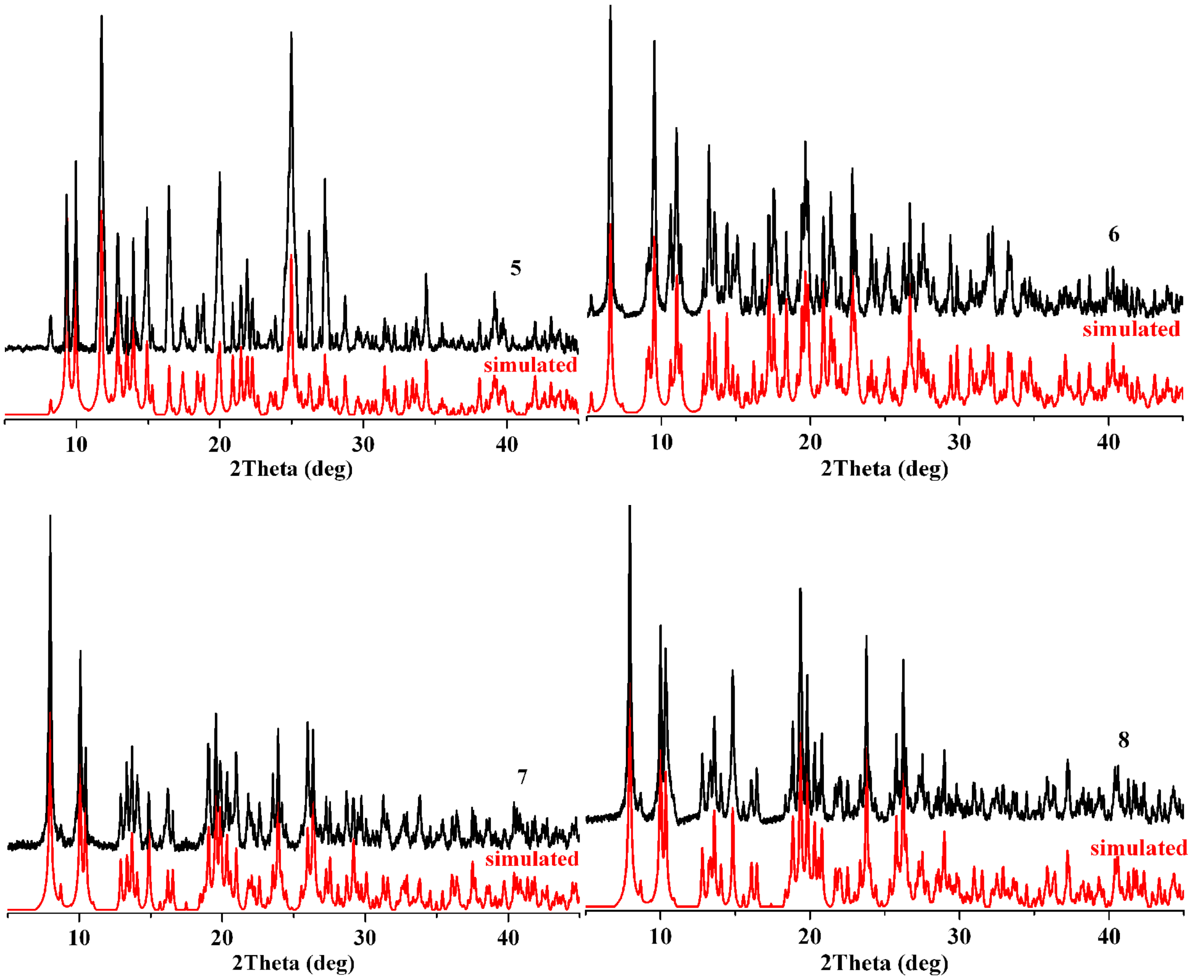

Figure S2b. PXRD patterns of compounds **5–8** at room temperature.

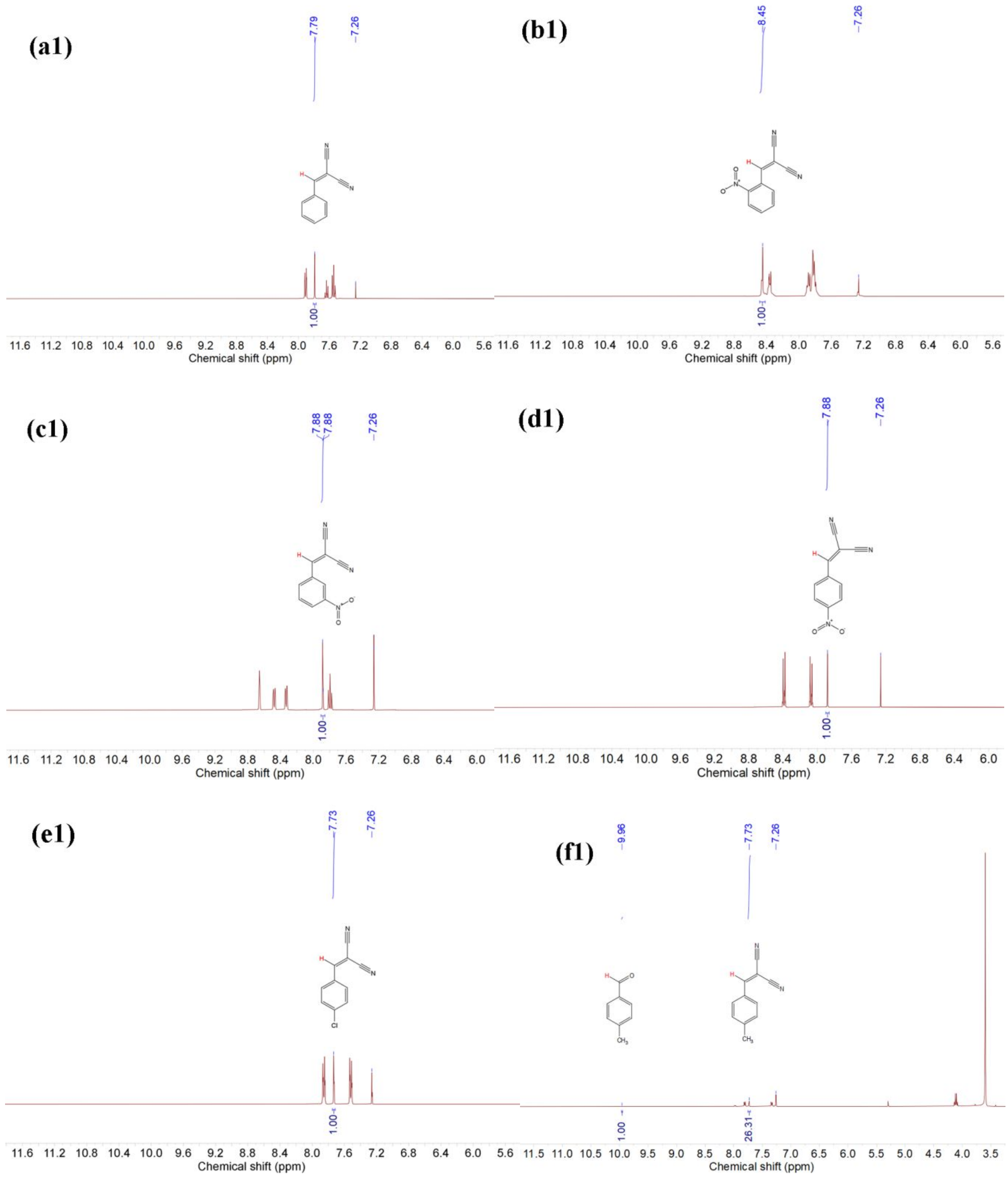

(a1)
7.79
7.26
1.00
Chemical shift (ppm)
(b1)
8.45
7.26
1.00
Chemical shift (ppm)
(c1)
7.88
7.88
7.26
1.00
Chemical shift (ppm)
(d1)
7.88
7.26
1.00
Chemical shift (ppm)
(e1)
7.73
7.26
1.00
Chemical shift (ppm)
(f1)
9.96
7.73
7.26
1.00
26.31
Chemical shift (ppm)

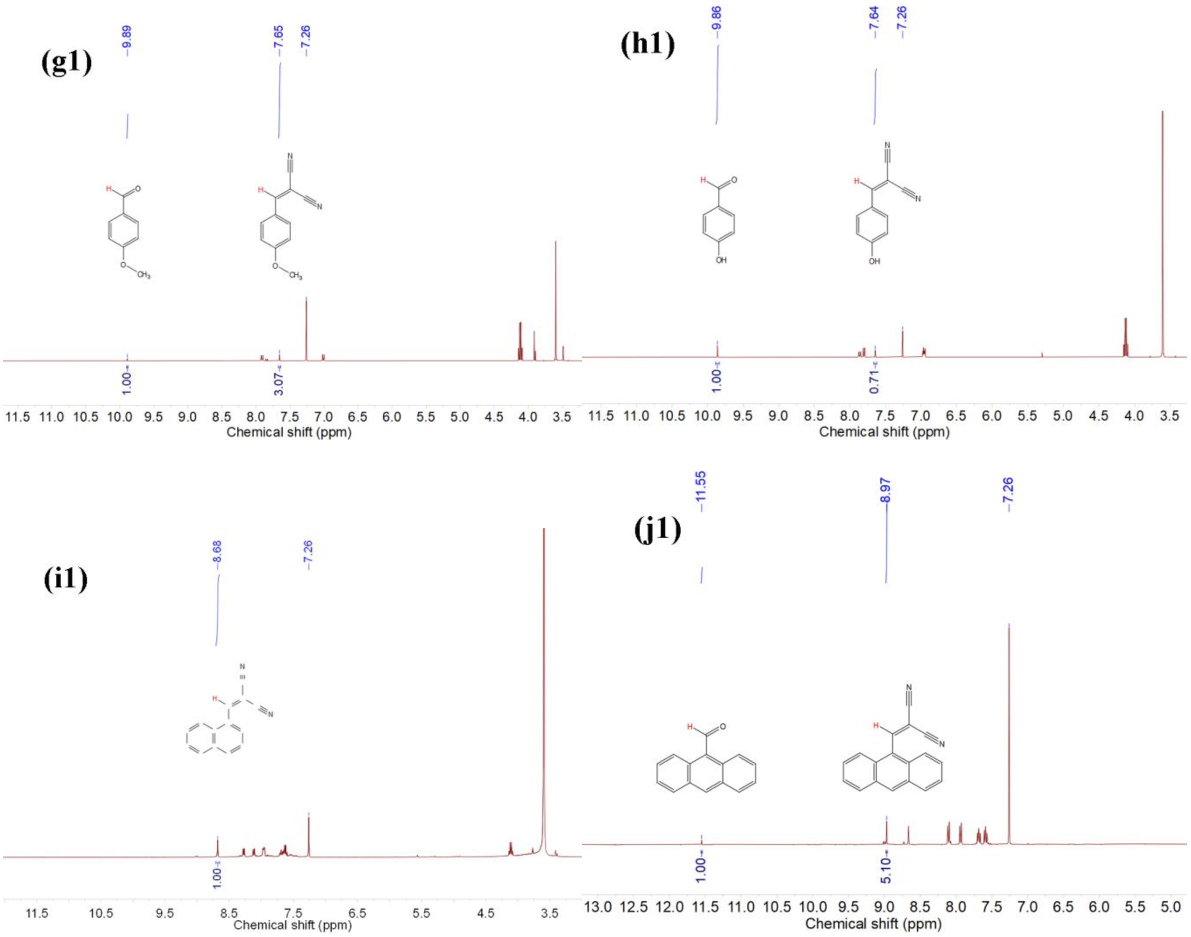

Figure S3. Examples of the integration of the ^{1}H NMR spectra of the reaction mixtures for determining the yields of condensation products formed from different aldehydes and malononitrile. Details for subfigures (a1)-(j1) are given below.

Calculation of the product yield based on the data of Figure S3

(a1) Conditions of Table 3, entry 6.

The C(=O)*H* signal of benzaldehyde (substrate) appears at δ 10.02 ppm, while 2-benzylidenemalononitrile (product) shows a characteristic signal at δ 7.79 ppm.

Total integration of both signals: unreacted benzaldehyde + 2-benzylidenemalononitrile = 0 + 1.00 = 1.00.

Percentage of the unreacted substrate: 0/1.00 = 0%

Conversion of benzaldehyde = yield of 2-benzylidenemalononitrile = 100 − 0 = 100%.

(b1) Conditions of Table S3, entry 2.

The C(=O)*H* signal of 2-nitrobenzaldehyde (substrate) appears at δ 10.40 ppm, while (2-nitrobenzylidene)malononitrile (product) shows a characteristic signal at δ 8.45 ppm.

Total integration of both signals: unreacted 2-nitrobenzaldehyde + (2-nitrobenzylidene)malononitrile = 0 + 1.00 = 1.00.

Percentage of the unreacted substrate: 0/1.00 = 0%

Conversion of 2-nitrobenzaldehyde = yield of (2-nitrobenzylidene)malononitrile = 100−0 = 100%.

(c1) Conditions of Table S3, entry 3.

The C(=O)*H* signal of 3-nitrobenzaldehyde (substrate) appears at δ 10.03 ppm, while (3-nitrobenzylidene)malononitrile (product) shows a characteristic signal at δ 7.88 ppm.

Total integration of both signals: unreacted 3-nitrobenzaldehyde + (3-nitrobenzylidene)malononitrile = 0 + 1.00 = 1.00.

Percentage of the unreacted substrate: 0/1.00 = 0%

Conversion of 3-nitrobenzaldehyde = yield of (3-nitrobenzylidene)malononitrile = 100 − 0 = 100%.

(d1) Conditions of Table S3, entry 4.

The C(=O)*H* signal of 4-nitrobenzaldehyde (substrate) appears at δ 10.15 ppm, while (4-nitrobenzylidene)malononitrile (product) shows a characteristic signal at δ 7.88 ppm.

Total integration of both signals: unreacted 4-nitrobenzaldehyde + (4-nitrobenzylidene)malononitrile = 0 + 1.00 = 1.00.

Percentage of the unreacted substrate: 0/1.00 = 0%

Conversion of 4-nitrobenzaldehyde = yield of (4-nitrobenzylidene)malononitrile = 100−0 = 100%.

(e1) Conditions of Table S3, entry 5.

The C(=O)*H* signal of 4-chlorobenzaldehyde (substrate) appears at δ 9.97 ppm, while (4-chlorobenzylidene)malononitrile (product) shows a characteristic signal at δ 7.73 ppm.

Total integration of both signals: unreacted 4-chlorobenzaldehyde + (4-chlorobenzylidene)malononitrile = 0+1.00 = 1.00.

Percentage of the unreacted substrate: 0/1.00 = 0%

Conversion of 4-chlorobenzaldehyde = yield of (4-chlorobenzylidene)malononitrile = 100−0 = 100%.

(f1) Conditions of Table S3, entry 6.

The –CH peak of 4-methylbenzaldehyde (substrate) appears at 9.96 ppm while that of (4-methylbenzylidene)malononitrile (product) can be seen at 7.73 ppm.

Total amount: unreacted substrate (4-methylbenzaldehyde) + formed product (4-methylbenzylidene)malononitrile = 1 + 26.31 = 27.31

Percentage of the unreacted substrate: 1/27.31 = 3.66%

Conversion of 4-methylbenzaldehyde = yield of (4-methylbenzylidene)malononitrile = 100−3.66 = 96.34%.

(g1) Conditions of Table S3, entry 7.

The –CH peak of 4-methoxybenzaldehyde (substrate) appears at 9.89 ppm while that of (4-methoxybenzylidene)malononitrile (product) can be seen at 7.65 ppm.

Total amount: unreacted substrate (4-methoxybenzaldehyde) + formed product (4-methoxybenzylidene)malononitrile = 1 + 3.07 = 4.07

Percentage of the unreacted substrate: 1/4.07 =24.6%

Conversion of 4-methoxybenzaldehyde = yield of (4-methoxybenzylidene)malononitrile = 100 − 24.6 = 75.4%.

(h1) Conditions of Table S3, entry 8.

The –CH peak of 4-hydroxybenzaldehyde (substrate) appears at 9.86 ppm while that of (4-hydroxybenzylidene)malononitrile (product) can be seen at 7.64 ppm.

Total amount: unreacted substrate (4-hydroxybenzaldehyde) + formed product (4-hydroxybenzylidene)malononitrile = 1+0.71 = 1.71

Percentage of the unreacted substrate: 1/1.71 = 58.5%

Conversion of 4-hydroxybenzaldehyde = yield of (4-hydroxybenzylidene)malononitrile = 100 − 58.5 = 41.5%.

(i1) Conditions of Table S3, entry 9.

The –CH peak of 1-naphthaldehyde (substrate) appears at 10.40 ppm while that of 2-(naphthalen-1-ylmethylidene)propanedinitrile (product) can be seen at 8.68 ppm.

Total amount: unreacted substrate (1-naphthaldehyde) + formed product 2-(naphthalen-1-ylmethylidene)propanedinitrile = 0 + 1.00 = 1.00

Percentage of the unreacted substrate: 0/1.00 = 0%

Conversion of 1-naphthaldehyde = yield of 2-(naphthalen-1-ylmethylidene)propanedinitrile = 100 − 0 = 100%.

(j1) Conditions of Table S3, entry 10.

The –CH peak of 9-anthraldehyde (substrate) appears at 11.55 ppm while that of 2-(anthracen-9-ylmethylene)malononitrile (product) can be seen at 8.97 ppm.

Total amount: unreacted substrate (9-anthraldehyde) + formed product 2-(anthracen-9-ylmethylene)malononitrile = 1+5.10=6.10

Percentage of the unreacted substrate: 1/6.10 = 16.4%

Conversion of 9-anthraldehyde = yield of 2-(anthracen-9-ylmethylene)malononitrile = 100 − 16.4 = 83.6%.

(a2)

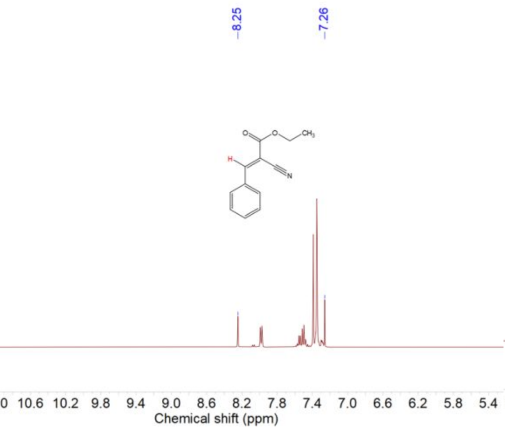

(b2)

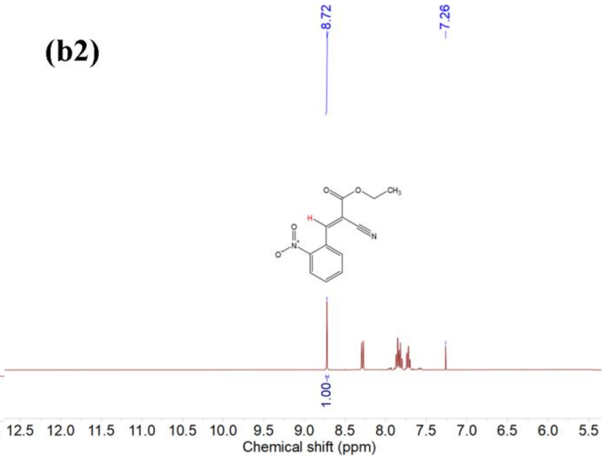

(c2)

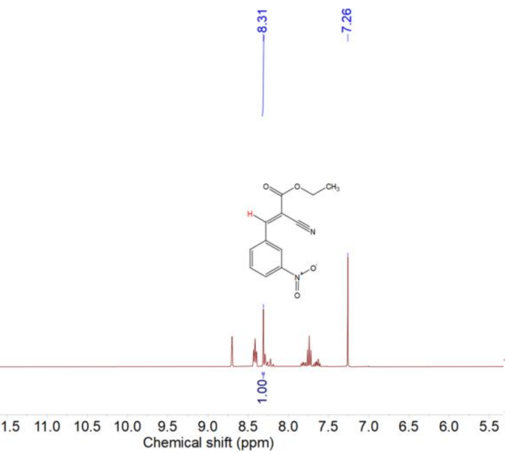

(d2)

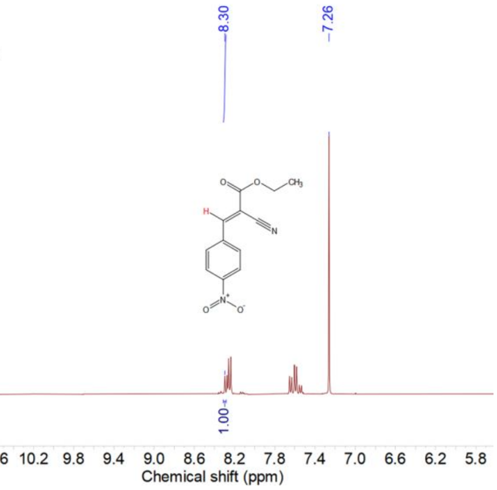

(e2)

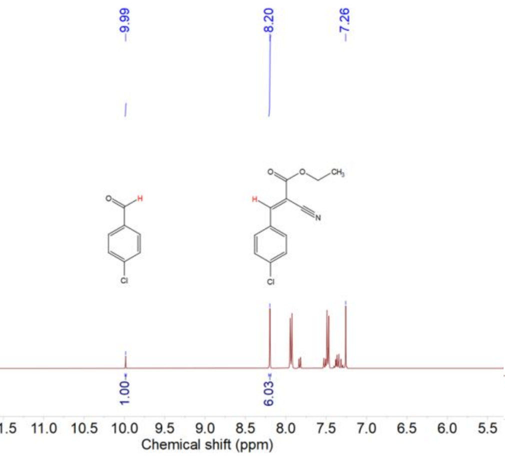

(f2)

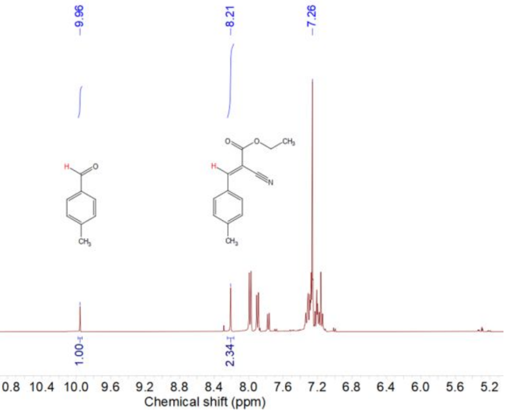

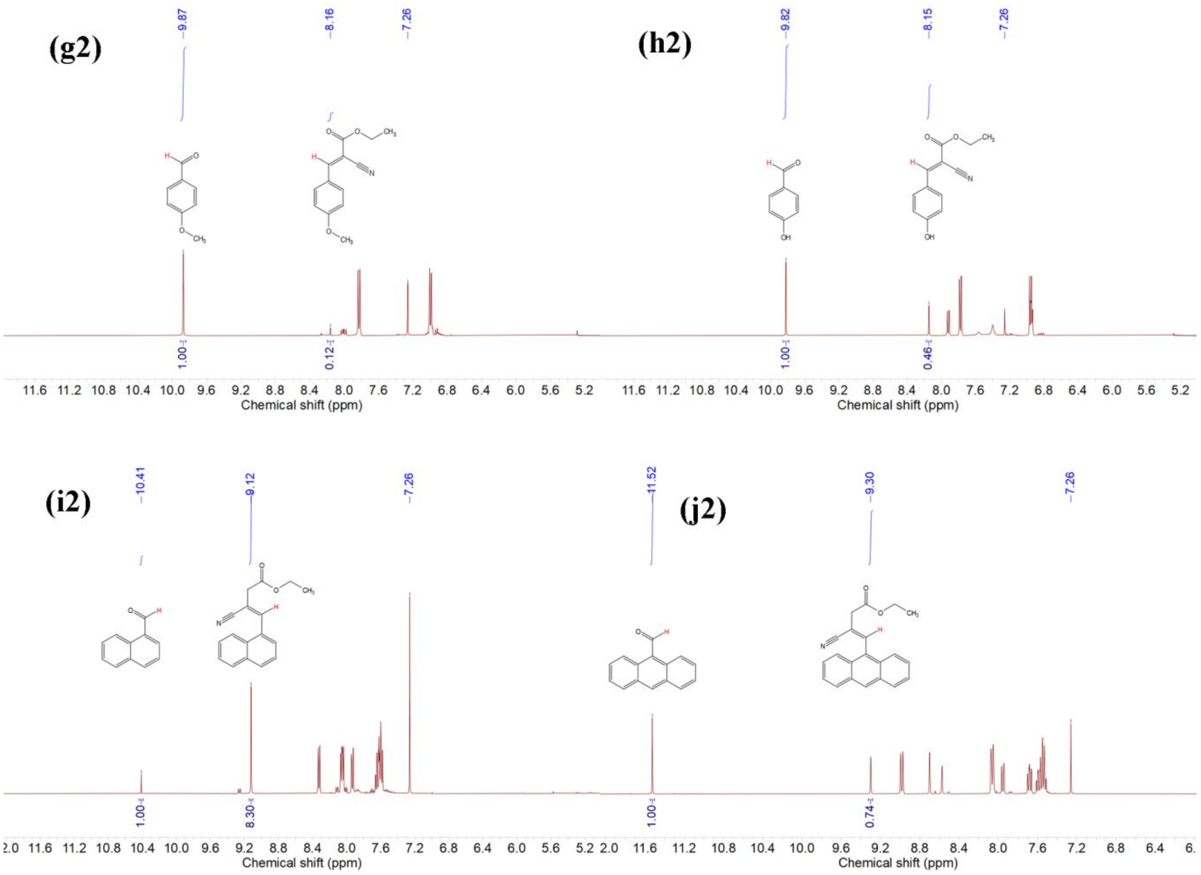

Figure S4. Examples of the integration of the ^{1}H NMR spectra of the reaction mixtures for determining the yields of condensation products formed from different aldehydes and ethyl cyanoacetate. Details for subfigures (a2)-(j2) are given below.

Calculation of the product yield based on the data of Figure S4

(a2) Conditions of Table S5, entry 4.

The C(=O)*H* signal of benzaldehyde (substrate) appears at δ 10.02 ppm, while ethyl-2-cyano-3-phenylacrylate (product) shows a characteristic signal at δ 8.25 ppm.

Total integration of both signals: unreacted benzaldehyde + product = 0 + 1.00 = 1.00.

Percentage of the unreacted substrate: 0/1.00 = 0%

Conversion of benzaldehyde = yield of product = 100 − 0 = 100%.

(b2) Conditions of Table S6, entry 2.

The C(=O)*H* signal of 2-nitrobenzaldehyde (substrate) appears at δ 10.40 ppm, while ethyl-2-cyano-3-(2-nitrophenyl)acrylate (product) shows a characteristic signal at δ 8.72 ppm.

Total integration of both signals: unreacted 2-nitrobenzaldehyde + product = 0 + 1.00 = 1.00.

Percentage of the unreacted substrate: 0/1.00 = 0%

Conversion of 2-nitrobenzaldehyde = yield of product = 100−0 = 100%.

(c2) Conditions of Table S6, entry 3.

The C(=O)*H* signal of 3-nitrobenzaldehyde (substrate) appears at δ 10.03 ppm, while ethyl-2-cyano-3-(3-nitrophenyl)acrylate (product) shows a characteristic signal at δ 8.31 ppm.

Total integration of both signals: unreacted 3-nitrobenzaldehyde + product = 0 + 1.00 = 1.00.
Percentage of the unreacted substrate: 0/1.00 = 0%
Conversion of 3-nitrobenzaldehyde = yield of product = 100 − 0 = 100%.

(d2) Conditions of Table S6, entry 4.
The C(=O)*H* signal of 4-nitrobenzaldehyde (substrate) appears at *δ* 10.15 ppm, while ethyl-2-cyano-3-(4-nitrophenyl)acrylate(product) shows a characteristic signal at *δ* 8.30 ppm.
Total integration of both signals: unreacted 4-nitrobenzaldehyde + product = 0 + 1.00 = 1.00.
Percentage of the unreacted substrate: 0/1.00 = 0%
Conversion of 4-nitrobenzaldehyde = yield of product = 100−0 = 100%.

(e2) Conditions of Table S6, entry 5.
The C(=O)*H* signal of 4-chlorobenzaldehyde (substrate) appears at *δ* 9.99 ppm, while ethyl-3-(4-chlorophenyl)-2-cyanoacrylate (product) shows a characteristic signal at *δ* 8.20 ppm.
Total integration of both signals: unreacted 4-chlorobenzaldehyde + 2-cyano-3-(4-chlorophenyl)-2-propenoic acid ethyl ester = 1+6.03 = 7.03.
Percentage of the unreacted substrate: 1/7.03 = 14.2%
Conversion of 4-chlorobenzaldehyde = yield of 2-cyano-3-(4-chlorophenyl)-2-propenoic acid ethyl ester = 100−14.2 = 85.8%.

(f2) Conditions of Table S6, entry 6.
The –CH peak of 4-methylbenzaldehyde (substrate) appears at 9.96 ppm while that of ethyl-2-cyano-3-(p-tolyl)acrylate(product) can be seen at 8.21 ppm.
Total amount: unreacted substrate (4-methylbenzaldehyde) + formed product = 1+2.34 = 3.34
Percentage of the unreacted substrate: 1/3.34 = 29.9%
Conversion of 4-methylbenzaldehyde = yield of product = 100−29.9 = 70.1%.

(g2) Conditions of Table S6, entry 7.
The –CH peak of 4-methoxybenzaldehyde (substrate) appears at 9.87 ppm while that of ethyl-2-cyano-3-(4-methoxyphenyl)acrylate(product) can be seen at 8.16 ppm.
Total amount: unreacted substrate (4-methoxybenzaldehyde) + formed product = 1 + 0.12 = 1.12
Percentage of the unreacted substrate: 1/1.12 = 89.3%
Conversion of 4-methoxybenzaldehyde = yield of product = 100 − 89.3 = 10.7%.

(h2) Conditions of Table S6, entry 8.
The –CH peak of 4-hydroxybenzaldehyde (substrate) appears at 9.82 ppm while that of ethyl-2-cyano-3-(4-hydroxyphenyl)acrylate(product) can be seen at 8.15 ppm.
Total amount: unreacted substrate (4-hydroxybenzaldehyde) + formed product = 1+0.46 = 1.46
Percentage of the unreacted substrate: 1/1.46 = 68.5%
Conversion of 4-hydroxybenzaldehyde = yield of product = 100 − 8.5 = 31.5%.

(i2) Conditions of Table S6, entry 9.
The –CH peak of 1-naphthaldehyde (substrate) appears at *δ* 10.41 ppm, while ethyl-3-cyano-4-(naphthalen-1-yl)but-3-enoate (product) shows a characteristic signal at *δ* 9.12 ppm.
Total integration of both signals: unreacted benzaldehyde + product = 1 + 8.30 = 9.30.

Percentage of the unreacted substrate: 1/9.30 = 10.8%
Conversion of benzaldehyde = yield of product = 100 − 10.8 = 89.2%.

(j2) Conditions of Table S6, entry 10.

The C(=O)*H* signal of 9-anthraldehyde (substrate) appears at *δ* 11.52 ppm, while ethyl-4-(anthracen-9-yl)-3-cyanobut-3-enoate (product) shows a characteristic signal at *δ* 9.30 ppm.
Total integration of both signals: unreacted benzaldehyde + product = 1 + 0.74 = 1.74.
Percentage of the unreacted substrate: 1/1.74 = 57.5%
Conversion of benzaldehyde = yield of product = 100 − 57.5 = 42.5%.

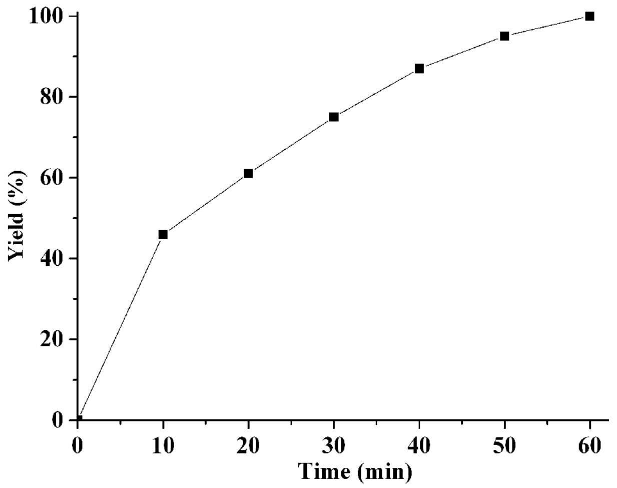

Figure S5. Accumulation of 2-benzylidenemalononitrile vs. time in the condensation reaction of benzaldehyde with malononitrile catalyzed by **5**. Reaction conditions are those of Table 3, entries 1−6.

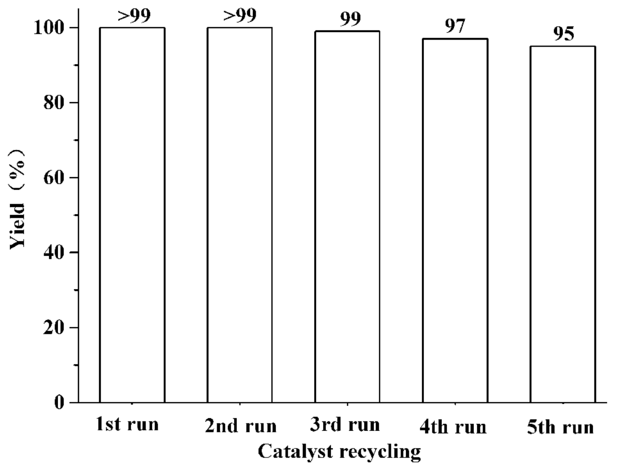

Figure S6. Catalyst recycling experiments in the condensation reaction of benzaldehyde with malononitrile catalyzed by **5**. Reaction conditions are those of Table 3, entry 6.

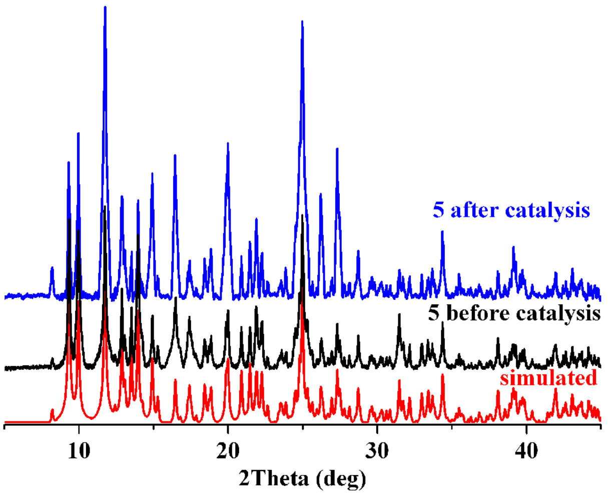

Figure S7. PXRD patterns of **5**: simulated (red), before (black) and after (blue) catalysis.

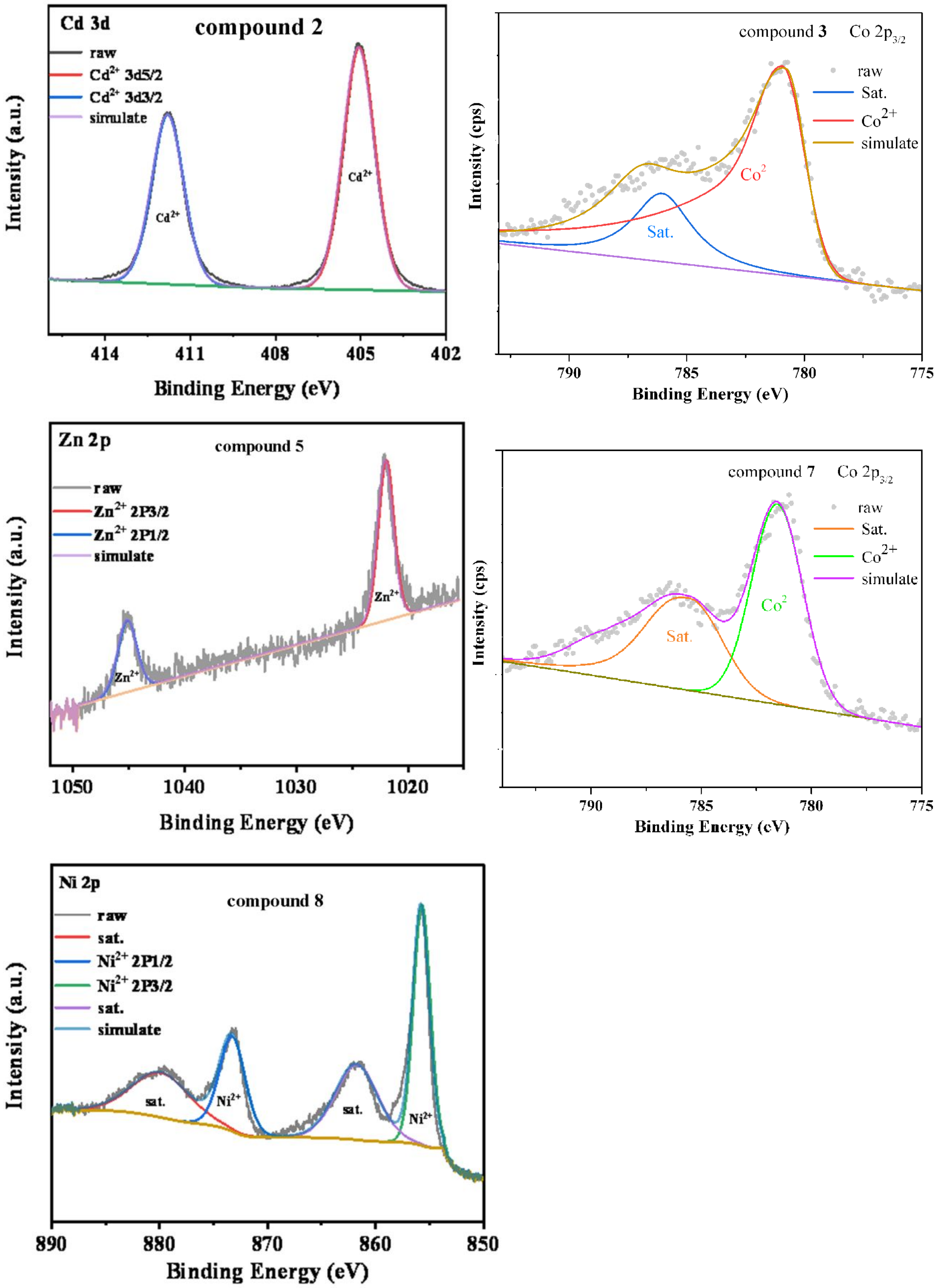

Figure S7. X-ray photoelectron spectra (XPS spectra) of **2**, **3**, **5**, **7**, and **8**.

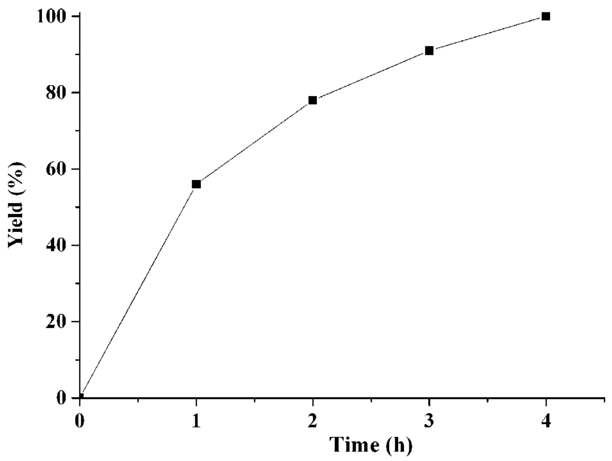

Figure S8. Accumulation of ethyl-2-cyano-3-phenylacrylate vs. time in the condensation reaction of benzaldehyde with ethyl cyanoacetate catalyzed by **5**. Reaction conditions are those of Table S5, entries 1−4.

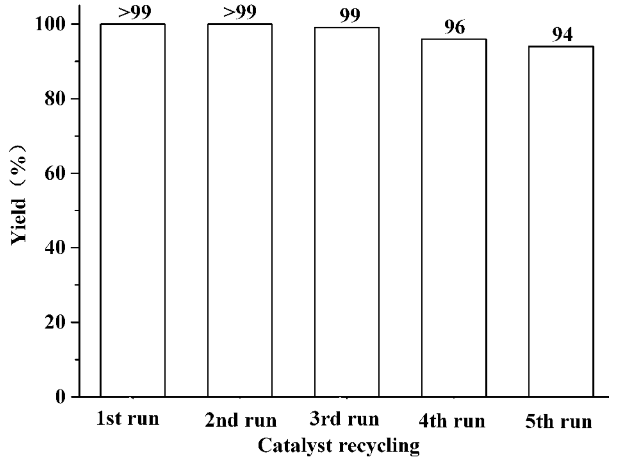

Figure S9. Catalyst recycling experiments in the condensation reaction of benzaldehyde with ethyl cyanoacetate catalyzed by **5**. Reaction conditions are those of Table S5, entry 4.

Table S1. Selected Bond Lengths [Å] and Angles [°] for the Compounds **1**–**8**[a].

**1**					
Zn(1)-O(1)	2.0712(13)	Zn(1)-O(1)i	2.0712(13)	Zn(1)-O(5)ii	2.0975(15)
Zn(1)-O(5)iii	2.0975(15)	Zn(1)-O(8)iv	2.1388(14)	Zn(1)-O(8)v	2.1388(14)
Zn(2)-O(2)	2.0300(15)	Zn(2)-O(6)ii	2.0410(16)	Zn(2)-O(7)iv	2.4554(16)
Zn(2)-O(8)iv	2.1017(14)	Zn(2)-N(1)	2.164(2)	Zn(2)-N(2)	2.0929(19)
O(1)-Zn(1)-O(1)i	176.62(9)	O(1)i-Zn(1)-O(5)ii	81.89(6)	O(5)ii-Zn(1)-O(1)	100.65(6)
O(5)ii-Zn(1)-O(5)iii	84.69(10)	O(1)i-Zn(1)-O(8)iv	87.05(5)	O(1)-Zn(1)-O(8)iv	90.92(6)
O(5)ii-Zn(1)-O(8)iv	163.87(6)	O(5)iii-Zn(1)-O(8)iv	85.88(6)	O(8)iv-Zn(1)-O(8)v	106.08(8)
O(2)-Zn(2)-O(6)ii	94.04(7)	O(2)-Zn(2)-N(2)	92.92(7)	O(6)ii-Zn(2)-N(2)	103.49(7)
O(2)-Zn(2)-O(8)iv	94.11(6)	O(6)ii-Zn(2)-O(8)iv	113.25(6)	O(8)iv-Zn(2)-N(2)	141.91(7)
O(2)-Zn(2)-N(1)	171.06(7)	O(6)ii-Zn(2)-N(1)	90.26(8)	N(1)-Zn(2)-N(2)	78.44(9)
O(8)iv-Zn(2)-N(1)	91.37(7)	O(2)-Zn(2)-O(7)iv	90.23(6)	O(6)ii-Zn(2)-O(7)iv	169.37(6)
O(7)iv-Zn(2)-N(2)	85.98(7)	O(8)iv-Zn(2)-O(7)iv	56.64(5)	N(1)-Zn(2)-O(7)iv	86.89(7)
**2**					
Cd(1)-O(2)	2.231(5)	Cd(1)-O(4)i	2.304(5)	Cd(1)-O(5)i	2.515(5)
Cd(1)-O(8)ii	2.247(6)	Cd(1)-N(1)	2.346(6)	Cd(1)-N(2)	2.284(6)
Cd(2)-O(1)	2.231(5)	Cd(2)-O(1)iii	2.230(5)	Cd(2)-O(4)i	2.273(5)
Cd(2)-O(4)iv	2.273(5)	Cd(2)-O(7)ii	2.263(5)	Cd(2)-O(7)v	2.263(5)
O(2)-Cd(1)-O(8)ii	94.8(2)	O(2)-Cd(1)-N(2)	97.8(2)	N(2)-Cd(1)-O(8)ii	105.2(2)
O(2)-Cd(1)-O(4)i	98.64(18)	O(4)i-Cd(1)-O(8)ii	112.69(18)	N(2)-Cd(1)-O(4)i	136.9(2)
N(1)-Cd(1)-O(2)	170.2(2)	N(1)-Cd(1)-O(8)ii	90.7(2)	N(2)-Cd(1)-N(1)	72.9(2)
N(1)-Cd(1)-O(4)i	86.7(2)	O(2)-Cd(1)-O(5)i	87.44(18)	O(5)i-Cd(1)-O(8)ii	166.79(16)
O(5)i-Cd(1)-N(2)	87.3(2)	O(4)i-Cd(1)-O(5)i	54.11(17)	O(5)i-Cd(1)-N(1)	89.1(2)
O(1)-Cd(2)-O(1)iii	175.5(3)	O(1)-Cd(2)-O(7)ii	98.2(2)	O(1)-Cd(2)-O(7)v	84.9(2)
O(7)v-Cd(2)-O(7)ii	93.2(3)	O(1)-Cd(2)-O(4)iv	89.57(19)	O(1)-Cd(2)-O(4)i	87.6(2)
O(4)iv-Cd(2)-O(7)v	83.75(19)	O(4)iv-Cd(2)-O(7)ii	171.34(18)	O(4)iv-Cd(2)-O(4)i	100.4(3)
**3**					
Co(1)-O(1)	2.096(2)	Co(1)-O(1)i	2.096(2)	Co(1)-O(8)ii	2.126(2)
Co(1)-O(8)iii	2.126(2)	Co(1)-N(2)iv	2.126(3)	Co(1)-N(1)v	2.126(3)
Co(2)-O(2)	1.988(3)	Co(2)-O(4)vi	2.274(3)	Co(2)-O(5)vi	2.050(3)
Co(2)-O(7)iii	2.158(3)	Co(2)-O(8)iii	2.223(3)	Co(2)-N(1)	2.104(3)
O(1)-Co(1)-N(2)iv	87.21(11)	O(1)-Co(1)-N(2)v	92.79(11)	O(1)-Co(1)-O(8)ii	89.29(10)
O(1)i-Co(1)-O(8)iii	90.71(10)	N(2)iv-Co(1)-O(8)iii	88.09(11)	N(2)iv-Co(1)-O(8)ii	91.91(11)
O(2)-Co(2)-O(5)vi	111.32(13)	N(1)-Co(2)-O(2)	93.32(12)	N(1)-Co(2)-O(5)vi	94.62(12)
O(2)-Co(2)-O(7)iii	157.57(11)	O(5)vi-Co(2)-O(7)iii	90.20(12)	N(1)-Co(2)-O(7)iii	91.07(12)
O(2)-Co(2)-O(8)iii	98.72(10)	O(5)vi-Co(2)-O(8)iii	137.48(11)	N(1)-Co(2)-O(8)iii	113.49(12)
O(8)iii-Co(2)-O(7)iii	59.54(10)	O(4)vi-Co(2)-O(2)	99.19(12)	O(5)vi-Co(2)-O(4)vi	60.53(11)
N(1)-Co(2)-O(4)vi	154.86(12)	O(7)iii-Co(2)-O(4)vi	85.63(12)	O(8)iii-Co(2)-O(4)vi	86.17(11)
**4**					
Zn(1)-O(1)	1.9696(17)	Zn(1)-O(4)i	2.043(2)	Zn(1)-O(5)i	2.382(2)
Zn(1)-O(7)ii	2.362(2)	Zn(1)-O(8)ii	2.1225(19)	Zn(1)-N(1)	2.0910(19)
Zn(2)-O(2)	2.1172(15)	Zn(2)-O(2)iii	2.1172(15)	Zn(2)-O(7)ii	2.1388(17)
Zn(2)-O(7)iv	2.1388(17)	Zn(2)-N(2)V	2.1175(18)	Zn(2)-N(2)vi	2.1175(18)
O(1)-Zn(1)-O(4)i	110.62(9)	O(1)-Zn(1)-N(1)	95.19(8)	N(1)-Zn(1)-O(4)i	95.09(8)
O(1)-Zn(1)-O(8)ii	152.13(8)	O(8)ii-Zn(1)-O(4)i	95.37(9)	N(1)-Zn(1)-O(8)ii	92.27(8)
O(1)-Zn(1)-O(7)ii	95.87(6)	O(7)ii-Zn(1)-O(4)i	134.56(8)	N(1)-Zn(1)-O(7)ii	119.26(7)
O(8)ii-Zn(1)-O(7)ii	57.39(7)	O(1)-Zn(1)-O(5)i	100.84(9)	O(4)i-Zn(1)-O(5)i	58.04(8)
N(1)-Zn(1)-O(5)i	152.19(8)	O(5)i-Zn(1)-O(8)ii	84.10(9)	O(5)i-Zn(1)-O(7)ii	81.71(8)

N(2)vi-Zn(2)-O(2)	92.29(7)	N(2)v-Zn(2)-O(2)	87.71(7)	O(7)ii-Zn(2)-O(2)	90.82(7)
O(7)iv-Zn(2)-O(2)	89.18(7)	N(2)v-Zn(2)-O(7)iv	89.94(8)	N(2)vi-Zn(2)-O(7)iv	90.06(8)
**5**					
Zn(1)-O(1)	1.975(3)	Zn(1)-O(5)i	2.014(3)	Zn(1)-O(7)ii	1.991(3)
Zn(1)-N(1)	2.049(3)				
O(1)-Zn(1)-O(7)ii	120.26(13)	O(1)-Zn(1)-O(5)i	134.39(13)	O(5)i-Zn(1)-O(7)ii	89.00(12)
O(1)-Zn(1)-N(1)	94.97(13)	N(1)-Zn(1)-O(7)ii	107.72(12)	N(1)-Zn(1)-O(5)i	109.26(12)
**6**					
Zn(1)-O(13)i	2.0639(14)	Zn(1)-O(17)	2.0674(14)	Zn(1)-O(18)	1.9694(14)
Zn(1)-N(1)	2.1755(17)	Zn(1)-N(3)ii	2.1550(17)	Zn(2)-O(1)	1.9382(14)
Zn(2)-O(16)iii	2.0018(14)	Zn(2)-O(18)	1.9493(14)	Zn(2)-N(2)	2.0447(17)
Zn(3)-O(4)i	2.0670(14)	Zn(3)-O(19)	2.0707(15)	Zn(3)-O(20)	1.9697(14)
Zn(3)-N(4)	2.1660(17)	Zn(3)-N(6)ii	2.1796(17)	Zn(4)-O(8)	1.9913(14)
Zn(4)-O(9)	1.9255(14)	Zn(4)-O(20)	1.9420(14)	Zn(4)-N(5)	2.0374(17)
O(18)-Zn(1)-O(13)i	102.05(6)	O(18)-Zn(1)-O(17)	102.00(6)	O(17)-Zn(1)-O(13)i	155.92(6)
O(18)-Zn(1)-N(3)ii	98.25(6)	O(13)i-Zn(1)-N(3)ii	90.91(6)	O(17)-Zn(1)-N(3)ii	84.41(6)
O(18)-Zn(1)-N(1)	96.15(6)	O(13)i-Zn(1)-N(1)	90.25(6)	O(17)-Zn(1)-N(1)	88.45(6)
N(3)ii-Zn(1)-N(1)	164.98(7)	O(18)-Zn(2)-O(1)	108.92(6)	O(1)-Zn(2)-O(16)iii	110.36(6)
O(18)-Zn(2)-O(16)ii	95.88(6)	O(1)-Zn(2)-N(2)	129.78(7)	O(18)-Zn(2)-N(2)	102.44(6)
N(2)-Zn(2)-O(16)ii	104.14(6)	O(20)-Zn(3)-O(4)i	96.98(6)	O(20)-Zn(3)-O(19)	99.09(6)
O(19)-Zn(3)-O(4)i	163.48(6)	N(4)-Zn(3)-O(20)	98.04(6)	N(4)-Zn(3)-O(4)i	93.69(6)
N(4)-Zn(3)-O(19)	87.85(7)	N(6)ii-Zn(3)-O(20)	97.45(6)	N(6)ii-Zn(3)-O(4)i	91.77(6)
N(6)ii-Zn(3)-O(19)	82.39(6)	N(4)-Zn(3)-N(6)ii	162.82(7)	O(9)-Zn(4)-O(20)	107.34(6)
O(8)-Zn(4)-O(9)	110.44(6)	O(20)-Zn(4)-O(8)	99.66(6)	N(5)-Zn(4)-O(9)	127.93(7)
N(5)-Zn(4)-O(20)	101.99(6)	N(5)-Zn(4)-O(8)	105.68(6)		
**7**					
Co(1)-O(1)	1.985(2)	Co(1)-O(4)i	2.0335(19)	Co(1)-O(7)ii	2.2080(18)
Co(1)-O(8)ii	2.2030(19)	Co(1)-N(1)	2.163(2)	Co(1)-N(2)iii	2.182(2)
Co(2)-O(5)	2.1142(17)	Co(2)-O(5)iv	2.1142(17)	Co(2)-O(7)	2.1367(18)
Co(2)-O(7)iv	2.1367(18)	Co(2)-N(3)	2.163(2)	Co(2)-N(3)iv	2.163(2)
O(1)-Co(1)-O(4)i	101.40(8)	O(1)-Co(1)-N(1)	95.28(8)	O(4)i-Co(1)-N(1)	90.57(8)
O(1)-Co(1)-N(2)iii	86.37(9)	N(2)iii-Co(1)-O(4)i	88.46(9)	N(2)iii-Co(1)-N(1)	178.23(9)
O(1)-Co(1)-O(8)ii	101.46(8)	O(4)i-Co(1)-O(8)ii	156.58(8)	O(8)ii-Co(1)-N(1)	92.32(8)
N(2)iii-Co(1)-O(8)ii	87.99(9)	O(7)ii-Co(1)-O(1)	160.72(8)	O(7)ii-Co(1)-O(4)i	97.88(7)
N(1)-Co(1)-O(7)ii	84.49(8)	N(2)iii-Co(1)-O(7)ii	94.17(8)	O(7)ii-Co(1)-O(8)ii	59.33(7)
O(5)-Co(2)-O(7)iv	88.37(7)	O(5)-Co(2)-O(7)	91.63(7)	O(5)-Co(2)-N(3)iv	90.46(7)
O(5)-Co(2)-N(3)	89.54(7)	N(3)-Co(2)-O(7)iv	92.95(7)	O(7)-Co(2)-N(3)	87.06(7)
O(7)-Co(2)-N(3)iv	92.94(7)				
**8**					
Ni(1)-O(1)	1.982(3)	Ni(1)-O(4)ii	2.034(3)	Ni(1)-O(6)iii	2.235(3)
Ni(1)-O(7)iii	2.193(3)	Ni(1)-N(1)	2.170(3)	Ni(1)-N(2)iv	2.186(3)
Ni(2)-O(5)	2.106(2)	Ni(2)-O(5)i	2.106(2)	Ni(2)-O(6)	2.144(2)
Ni(2)-O(6)i	2.144(2)	Ni(2)-N(3)	2.170(3)	Ni(2)-N(3)i	2.170(3)
O(1)-Ni(1)-O(4)ii	102.14(14)	O(1)-Ni(1)-O(6)iii	160.85(14)	O(1)-Ni(1)-O(7)iii	102.01(14)
O(1)-Ni(1)-N(1)	95.76(14)	O(1)-Ni(1)-N(2)iv	85.92(15)	O(6)iii-Ni(1)-O(4)ii	97.00(11)
O(7)iii-Ni(1)-O(4)ii	155.29(12)	N(1)-Ni(1)-O(4)ii	90.64(13)	N(2)iv-Ni(1)-O(4)ii	88.82(15)
O(6)iii-Ni(1)-O(7)iii	58.87(11)	O(6)iii-Ni(1)-N(1)	84.61(12)	O(7)iii-Ni(1)-N(1)	92.07(13)
N(2)iv-Ni(1)-N(1)	178.30(14)	O(6)iii-Ni(1)-N(2)iv	93.86(13)	O(7)iii-Ni(1)-N(2)iv	87.76(13)
O(1)ii-Mn(2)-O(8)	168.35(7)	O(12)-Mn(2)-O(8)	92.50(8)	O(1)ii-Mn(2)-O(12)	91.06(8)
O(5)-Ni(2)-O(6)	91.48(11)	O(6)i-Ni(2)-O(5)	88.52(11)	O(5)-Ni(2)-N(3)	90.24(12)

O(5)-Ni(2)-N(3)i	89.77(12)	O(6)i-Ni(2)-N(3)	87.65(12)	O(6)-Ni(2)-N(3)	92.35(12)

[a]Symmetry transformations used to generate equivalent atoms: i –*x*+2, *y*, –*z*+3/2; ii *x*+1/2, *y*–1/2, *z*; iii –*x*+3/2, *y*–1/2, –*z*+3/2; iv *x*+1/2, *y*+1/2, *z* ; v –*x*+3/2, *y*+1/2, –*z*+3/2 for **1**; i *x*, *y*–1/2, *z*+1/2; ii *x*, *y*–1/2, *z*–1/2; iii –*x*+1, –*y*, –*z*; iv –*x*+1, –*y*+1/2, *z*+1/2; v –*x*+1, –*y*+1/2, *z*–1/2 for **2**; i –*x*+1, –*y*, –*z*; ii *x*–1/2, –*y*+1/2, *z*–1/2; iii –*x*+3/2, *y*–1/2, –*z*+1/2; iv *x*, *y*, *z*–1; v –*x*+1, –*y*, –*z*+1; vi *x*+1, *y*, *z* for **3**; i *x*–1, *y*, *z*; ii –*x*+1/2, *y*–1/2, –*z*+3/2; iii –*x*+1, –*y*, –*z*+2; iv *x*+1/2, –*y*+1/2, *z*+1/2; v *x*, *y*, *z*+1; vi –*x*+1, –*y*, –*z*+1 for **4**; i *x*–1/2, –*y*+1/2, –*z*+1; ii –*x*+1, –*y*+1, –*z*+1 for **5;** i –*x*+1, –*y*, –*z*+1; ii *x*, *y*–1, *z*; iii *x*+1, *y*, *z*+1 for **6**; i *x*+1/2, –*y*+3/2, *z*+1/2; ii –*x*+3/2, *y*+1/2, –*z*+1/2; iii *x*, *y*, *z*–1; iv –*x*+1, –*y*+1, –*z* for **7**; i –*x*+1, –*y*+ 1, –*z*+2; ii *x*–1/2, –*y*+3/2, *z*–1/2; iii –*x*+1/2, *y*+1/2, –*z*+3/2; iv *x*, *y*, *z*+1 for **8**.

Table S2. Hydrogen Bonds in Crystal Packing [Å, °] of **1**–**3** and **5**–**7**.

Complexes	D-H…A	*d*(D-H)	*d*(H…A)	*d*(D…A)	∠DHA	Symmetry code
**1**	O(10)-H(4W)···O(1)	0.850	2.490	3.011	120.44	-*x*+3/2, *y*+1/2, -*z*+3/2
**2**	O(9)-H(1W)···O(3)	0.869	2.196	3.017	157.55	
	O(9)-H(2W)···O(7)	0.870	2.340	3.077	142.64	
	O(10)-H(3W)···O(5)	0.869	2.423	2.873	112.73	*x*, *y*-1/2, *z*+1/2
	O(10)-H(4W)···O(9)	0.870	2.135	2.806	133.46	
**3**	O(9)-H(1W)···O(3)	0.850	2.062	2.912	179.45	*x*-1/2, -*y*+1/2, *z*+1/2
	O(9)-H(2W)···O(5)	0.850	2.108	2.958	179.45	
**5**	N(2)-H(1)···O(5)	0.860	2.161	2.964	155.25	*x*, -*y*+1/2, *z*+1/2
**6**	O(17)-H(1W)···O(12)	0.870	1.823	2.656	159.61	*x*+1, *y*, *z*+1
	O(17)-H(2W)···O(16)	0.870	2.069	2.749	134.48	*x*+1, *y*, *z*+1
	O(19)-H(3W)···O(5)	0.870	1.814	2.616	152.40	
	O(19)-H(4W)···O(8)	0.870	1.993	2.705	138.24	
	O(21)-H(5W)···O(15)	0.870	2.078	2.920	162.61	-*x*+1, -*y*+1, -*z*
	O(21)-H(6W)···O(2)	0.870	1.900	2.764	171.64	
	O(22)-H(7W)···O(10)	0.870	1.931	2.790	169.06	-*x*+1, -*y*+1, -*z*
	O(22)-H(8W)···O(7)	0.870	2.130	2.980	165.57	
**7**	O(9)-H(1W)···O(2)	0.870	1.973	2.816	163.03	
	O(9)-H(2W)···O(8)	0.870	2.431	3.300	175.87	-*x*+2, -*y*+1, -*z*+1
**8**	O(9)-H(9A)···O(2)	0.850	1.978	2.817	169.13	

Table S3. Substrate Scope in the Zn-catalyzed Condensation Reaction of Different Aldehyde Substrates with Malononitrile.[a]

Entry	Aldehyde substrate	Product yield, %[b]
1	benzaldehyde	99
2	2-nitrobenzaldehyde	99
3	3-nitrobenzaldehyde	99
4	4-nitrobenzaldehyde	99
5	4-chlorobenzaldehyde	99
6	4-methylbenzaldehyde	96
7	4-methoxybenzaldehyde	75
8	4-hydroxybenzaldehyde	42
9	1-naphthaldehyde	99
10	9-anthraldehyde	84

[a]Conditions: aldehyde (0.5 mmol), malononitrile (1.0 mmol), catalyst **5** (2.0 mol.%), CH_3OH (1.0 mL), 25 °C, 1 h. [b]Yield on the basis of ^{1}H NMR analysis: [moles of product per mol of aldehyde substrate]×100%.

Table S4. Comparison of Various Catalysts in the Condensation Reaction between Benzaldehyde and Malononitrile.[a]

Entry	Catalyst	Catalyst (mol%)	Solvent	Time (h)	Temp. (°C)	Yield (%)	TON	TOF	Ref.
1	$[Zn(\mu_3\text{-cpbda})(Hbpa)]_n \cdot 4nH_2O$	2	MeOH	1	25	99	50	50	This work
2	$[Zn_4(\mu_3\text{-cpbda})_2(\mu\text{-OH})_2(\mu\text{-dpey})_3(H_2O)_2]_n \cdot 2nH_2O$	2	MeOH	1	25	99	50	50	This work
3	$Zn_3(OH)(ATTCA)_2(H_2O)] \cdot C_2H_6NH_2 \cdot 4DMF \cdot H_2O$	10	DCM	5	25	94	10	2	44
4	$[Zn_3(L)_2(\mu_2\text{-OH})_2]_n$	4	H_2O	8	90	78	20	2.5	45
5	$\{[Zr_6(\mu_3\text{-O})_4(\mu_3\text{-OH})_4(OH)_4(H_2O)_4(DCBA)_2] \cdot 5DMF \cdot 3H_2O\}$	10	DCM	5	25	89	9	2	47
6	$\{[Cd(Py_2TTz)(2\text{-NH}_2\text{-BDC})] \cdot (DMF) \cdot 0.5(H_2O)\}_n$	2	free	6	60	99.8	50	8	48

[a]Abbreviations: H_3ATTCA = 2-amino[1,1:3,1-terphenyl]-4,4,5-tricarboxylic acid; H_2L = 2-(hydroxymethyl)-1H-benzo[d]imidazole-5-carboxylic acid; H_4DCBA = 4″-6′-diamino-5′,5″-bis(4-carboxyphenyl)-[1,1′:3′,1″:3″,1‴-quaterphenyl]-4,4‴-dicarboxylic acid; Py_2TTz = 2,5-bis(4-pyridyl)thiazolo[5,4-*d*]thiazole; 2-NH_2-BDC = 2-amino-1,4-benzenedicarboxylate.

Table S5. CP-catalyzed Condensation of Benzaldehyde with Ethyl Cyanoacetate.[a]

Entry	Catalyst	Reaction time/h, T/°C	Catalyst loading, mol%	Solvent	Product yield, %[b]
1	**5**	1, 40	2.0	CH_3OH	56
2	**5**	2, 40	2.0	CH_3OH	78
3	**5**	3, 40	2.0	CH_3OH	91
4	**5**	4, 40	2.0	CH_3OH	99
5	**5**	4, 40	1.0	CH_3OH	94
6	**5**	4, 30	2.0	CH_3OH	74
7	**5**	4, 40	2.0	H_2O	60
8	**5**	4, 40	2.0	C_2H_5OH	56
9	**5**	4, 40	2.0	CH_3CN	38
10	**5**	4, 40	2.0	$CHCl_3$	32
11	**1**	4, 40	2.0	CH_3OH	86
12	**2**	4, 40	2.0	CH_3OH	87
13	**3**	4, 40	2.0	CH_3OH	83
14	**4**	4, 40	2.0	CH_3OH	80
15	**6**	4, 40	2.0	CH_3OH	99
16	**7**	4, 40	2.0	CH_3OH	81
17	**8**	4, 40	2.0	CH_3OH	78
18	blank	4, 40	–	CH_3OH	3
19	$ZnCl_2$	4, 40	2.0	CH_3OH	12
20	H_3cpbda	4, 40	2.0	CH_3OH	17

[a] Conditions: benzaldehyde (0.5 mmol), ethyl cyanoacetate (1.0 mmol), catalyst (1−2 mol%), solvent (1.0 mL), 40 °C. [b]Yield on the basis of ^{1}H NMR analysis: [moles of product per mol of aldehyde substrate]×100%.

Table S6. Substrate Scope in the Zn-catalyzed Condensation Reaction of Different Aldehyde Substrates with Ethyl Cyanoacetate.[a]

Entry	Aldehyde Substrate	Product yield, %[b]
1	benzaldehyde	99
2	2-nitrobenzaldehyde	99
3	3-nitrobenzaldehyde	99
4	4-nitrobenzaldehyde	99
5	4-chlorobenzaldehyde	86
6	4-methylbenzaldehyde	70
7	4-methoxybenzaldehyde	11
8	4-hydroxybenzaldehyde	32
9	1-naphthaldehyde	89
10	9-anthraldehyde	42

[a] Conditions: aldehyde (0.5 mmol), ethyl cyanoacetate (1.0 mmol), catalyst (1−2 mol%), solvent (1.0 mL), 40 °C, 4 h. [b]Yield on the basis of ^{1}H NMR analysis: [moles of product per mol of aldehyde substrate]×100%.
